# Supplementary material for: Design, Synthesis, In Vitro, and In Silico Insights of 5-(Substituted benzylidene)-2-phenylthiazol-4(5H)-one Derivatives: A Novel Class of Anti-Melanogenic Compounds
Source: Molecules. 2023 Apr 7;28(8):3293. doi: 10.3390/molecules28083293 (PMC10144242; doi:10.3390/molecules28083293)
Supplement: Supplementary file 1 [file molecules-28-03293-s001.zip › molecules-2316492-supplementary.pdf]

Supplementary Materials

for

Design, Synthesis, In Vitro, and In Silico Insights of 5-(Substituted benzylidene)-2-phenylthiazol-4(5*H*)-one Derivatives: A Novel Class of Anti-Melanogenic Compounds

Dahye Yoon [1,†], Min Kyung Kang [1,†], Hee Jin Jung [2,†], Sultan Ullah [3], Jieun Lee [1], Yeongmu Jeong [1], Sang Gyun Noh [2], Dongwan Kang [4], Yujin Park [4], Pusoon Chun [5], Hae Young Chung [2] and Hyung Ryong Moon [1,*]

[1] Department of Manufacturing Pharmacy, College of Pharmacy, Pusan National University, Busan 46241, Republic of Korea; dahae0528@pusan.ac.kr (D.Y.); kmk87106@pusan.ac.kr (M.K.K.); yijiun@pusan.ac.kr (J.L.); dassabn@pusan.ac.kr (Y.J.)
[2] Department of Pharmacy, College of Pharmacy, Pusan National University, Busan 46241, Republic of Korea; hjjung2046@pusan.ac.kr (H.J.J.); rskrsk92@pusan.ac.kr (S.G.N.); hyjung@pusan.ac.kr (H.Y.C.)
[3] Department of Molecular Medicine, The Herbert Wertheim UF Scripps Institute for Biomedical Innovation & Technology, Jupiter, FL 33458, USA; sultanullahf@ufl.edu
[4] Department of Medicinal Chemistry, New Drug Development Center, Daegu-Gyeongbuk Medical Innovation Foundation, Daegu 41061, Republic of Korea; kdw4106@kmedihub.re.kr (D.K.); pyj1016@kmedihub.re.kr (Y.P.)
[5] College of Pharmacy and Inje Institute of Pharmaceutical Sciences and Research, Inje University, Gimhae 50834, Republic of Korea; pusoon@inje.ac.kr
* Correspondence: mhr108@pusan.ac.kr; Tel.: +82 51 510 2815; Fax: +82 51 513 6754
† These authors contributed equally to this work.

Contents

Figure S1. ^{1}H NMR spectrum of compound **1**……….…………………………………………4

Figure S2. ^{13}C NMR spectrum of compound **1**……….…………………………..….…………5

Figure S3. LRMS spectrum of compound **1**…..……………………………….. ……...……6

Figure S4. ^{1}H NMR spectrum of compound **2**………..……………………………….…………7

Figure S5. ^{13}C NMR spectrum of compound **2**………..………….…..…………………….8

Figure S6. Proton-coupled ^{13}C NMR spectrum of compound **2**………..……..……..………9

Figure S7. LRMS spectrum of compound **2**………..……………..……….…….……..…10

Figure S8. ^{1}H NMR spectrum of compound **3**…….……………………………………..…...11

Figure S9. ^{13}C NMR spectrum of compound **3**……..……………..……………………….12

Figure S10. LRMS spectrum of compound **3**…..……..…………….………………..…..13

Figure S11. ^{1}H NMR spectrum of compound **4**…..…………..…………………………..…..14

Figure S12. ^{13}C NMR spectrum of compound **4**…………..……………….……………..…15

Figure S13. LRMS spectrum of compound **4**………………..…………………………...…...16

Figure S14. ^{1}H NMR spectrum of compound **5**……….…………………………..…..…….......17

Figure S15. ^{13}C NMR spectrum of compound **5**……..……………......………..……….…18

Figure S16. LRMS spectrum of compound **5**…….…………………………….……………19

Figure S17. ^{1}H NMR spectrum of compound **6**…….………………………...………….…...20

Figure S18. ^{13}C NMR spectrum of compound **6**……….....…………………………………..…21

Figure S19. LRMS spectrum of compound **6**…………..….. …….……………...…...22

Figure S20. ^{1}H NMR spectrum of compound **7**……..……………………...………...…...23

Figure S21. ^{13}C NMR spectrum of compound **7**…….…...………………………………..…24

Figure S22. LRMS spectrum of compound **7**…….…………………..……………….…..…..25

Figure S23. ^{1}H NMR spectrum of compound **8** (in DMSO-d_6)………………………...…..26

Figure S24. ^{13}C NMR spectrum of compound **8** (in DMSO-d_6)…….……………………….………27

Figure S25. LRMS spectrum of compound **8**……..………………..…..………………..…..…..28

Figure S26. ^{1}H NMR spectrum of compound **9**……..……………………....……………..…..29

Figure S27. ^{13}C NMR spectrum of compound **9**……..…...………………..……………………..30

Figure S28. LRMS spectrum of compound **9**……..……………………………………..…...…..31

Figure S29. ^{1}H NMR spectrum of compound **10**………..………………..……..……...…..32

Figure S30. ^{13}C NMR spectrum of compound **10**……......…………………….………….33

Figure S31. LRMS spectrum of compound **10**……..…………………………………....…..34

Figure S32. ^{1}H NMR spectrum of compound **11**……………………………..…...…..35

Figure S33. ^{13}C NMR spectrum of compound **11**………..……………………….………….36

Figure S34. LRMS spectrum of compound **11**………………………………………....…..37

Figure S35. ^{1}H NMR spectrum of compound **12**……...………………….....…………....…...38

Figure S36. ^{13}C NMR spectrum of compound **12**……......…………………….………….39

Figure S37. LRMS spectrum of compound **12**………..………………………..……...40

Figure S38. ^{1}H NMR spectrum of compound **13**....………………………...…………....…..41

Figure S39. ^{13}C NMR spectrum of compound **13**……....……………………………..…...42

Figure S40. LRMS spectrum of compound **13**…………….…………………….....……43

Figure S41. ^{1}H NMR spectrum of compound **14**....………….....……………....………....…..44

Figure S42. ^{13}C NMR spectrum of compound **14**……....…………………………..……...45

Figure S43. LRMS spectrum of compound **14**……………….…………………..…...……46

Figure S44. ^{1}H NMR spectrum of compound **8** (in $CDCl_3$) reported in other article………...47

Figure S45. ^{13}C NMR spectrum of compound **8** (in $CDCl_3$) reported in other article ……....…48

Figure S46. ^{1}H NMR spectrum of compound **8** in $CDCl_3$………………......……...……...…..49

Figure S47. ^{13}C NMR spectrum of compound **8** in $CDCl_3$…...……………………………50

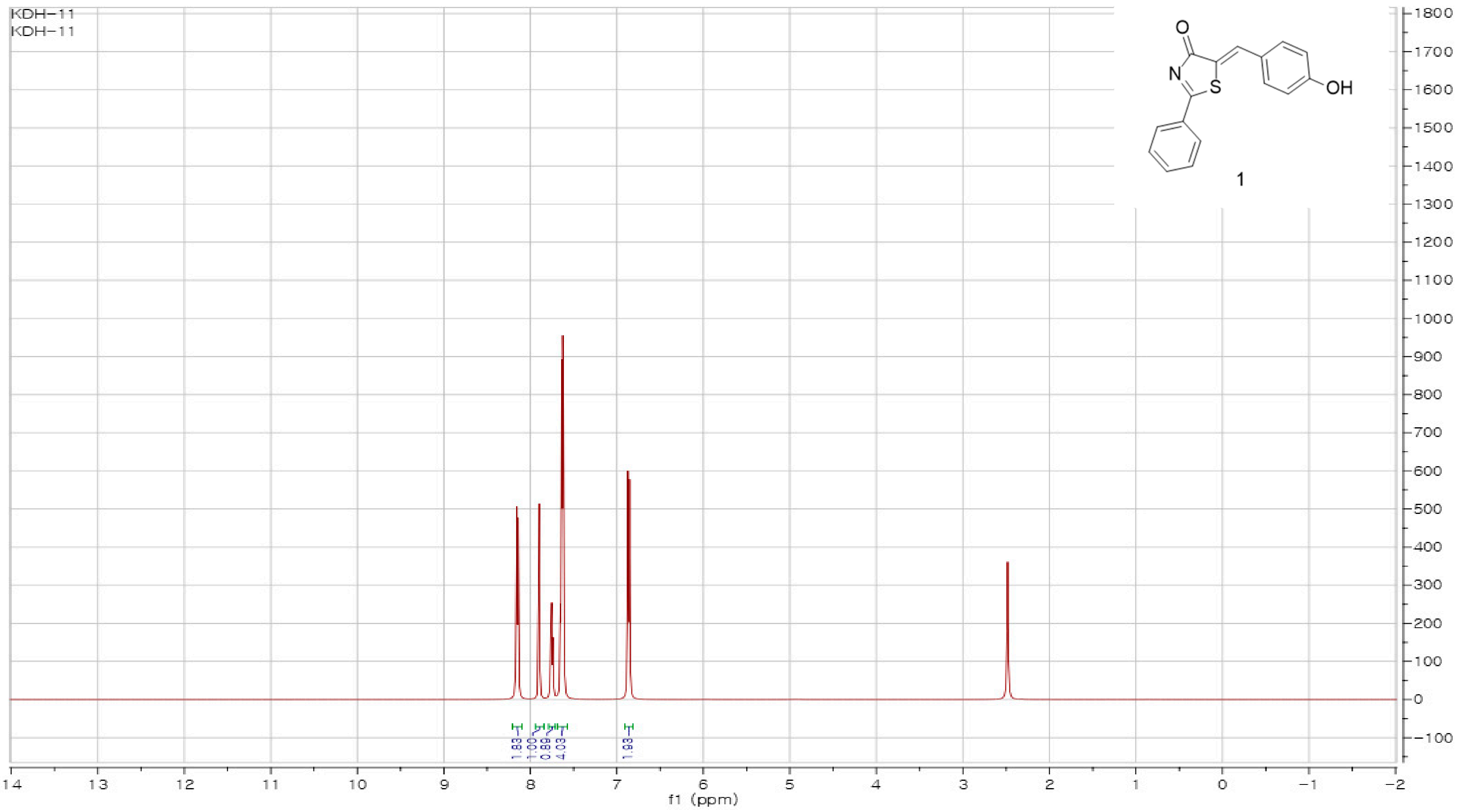

Figure S1. ^{1}H NMR spectrum of compound **1**.

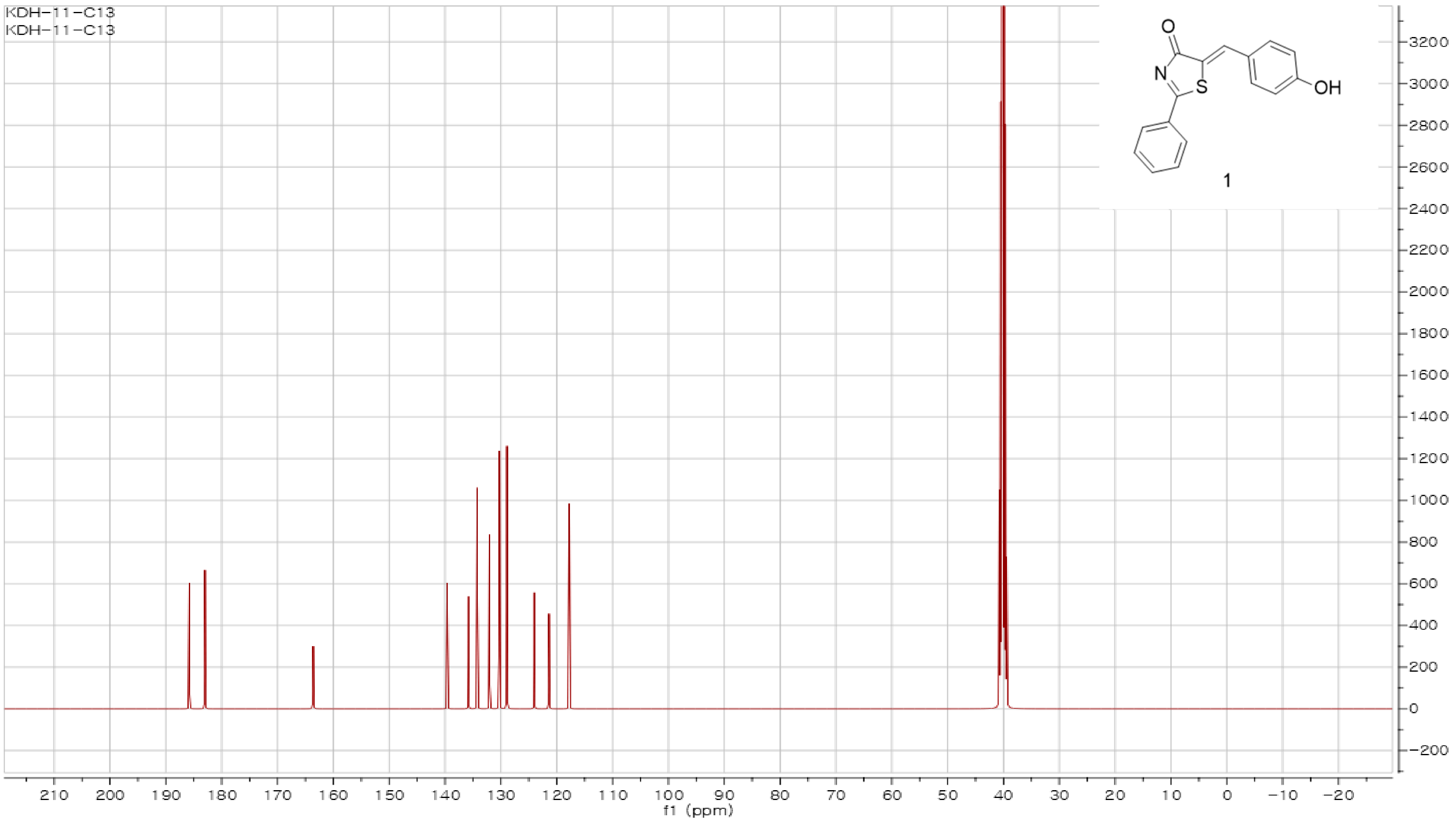

Figure S2. ^{13}C NMR spectrum of compound **1**.

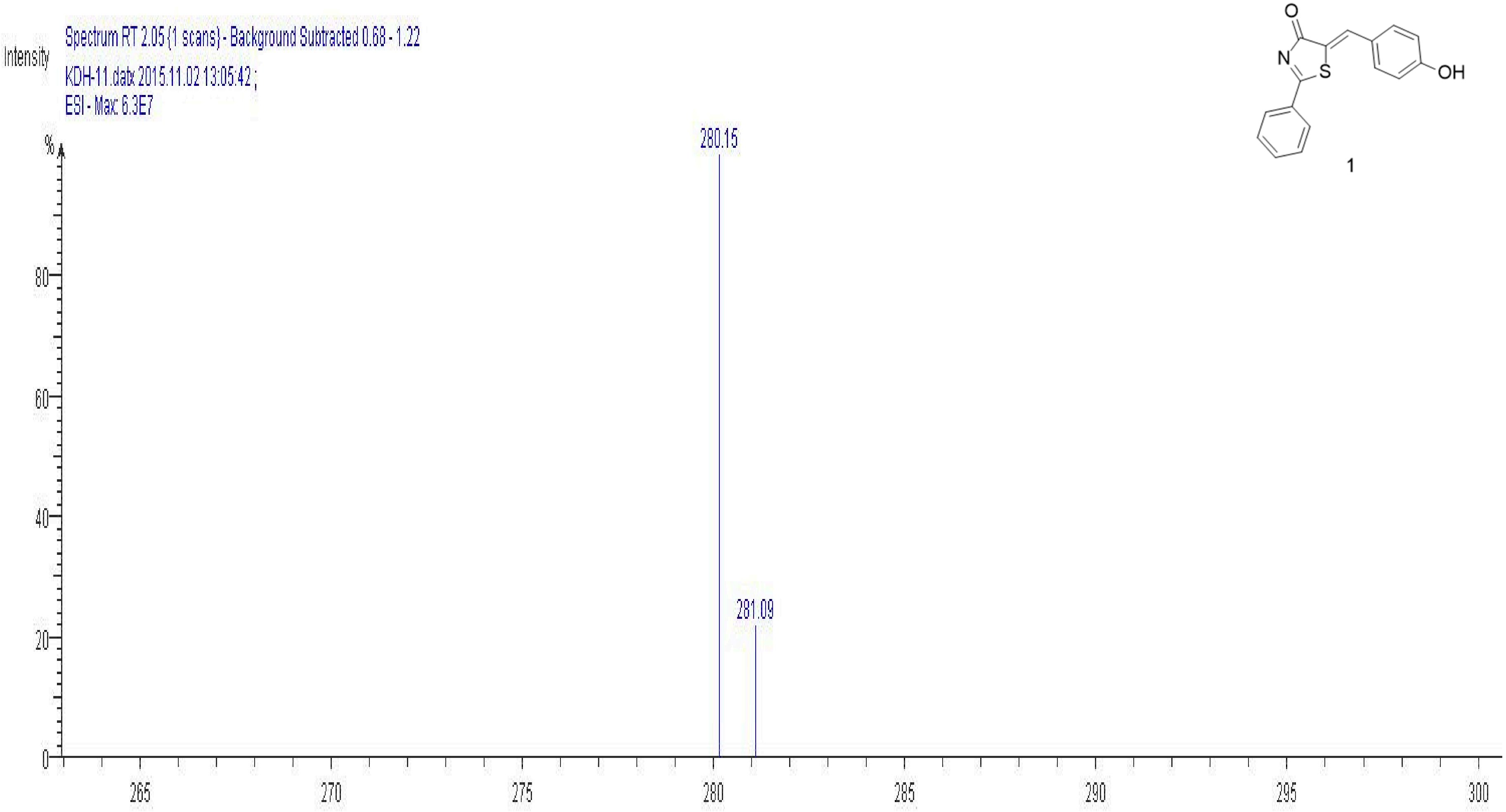

Figure S3. LRMS spectrum of compound **1**.

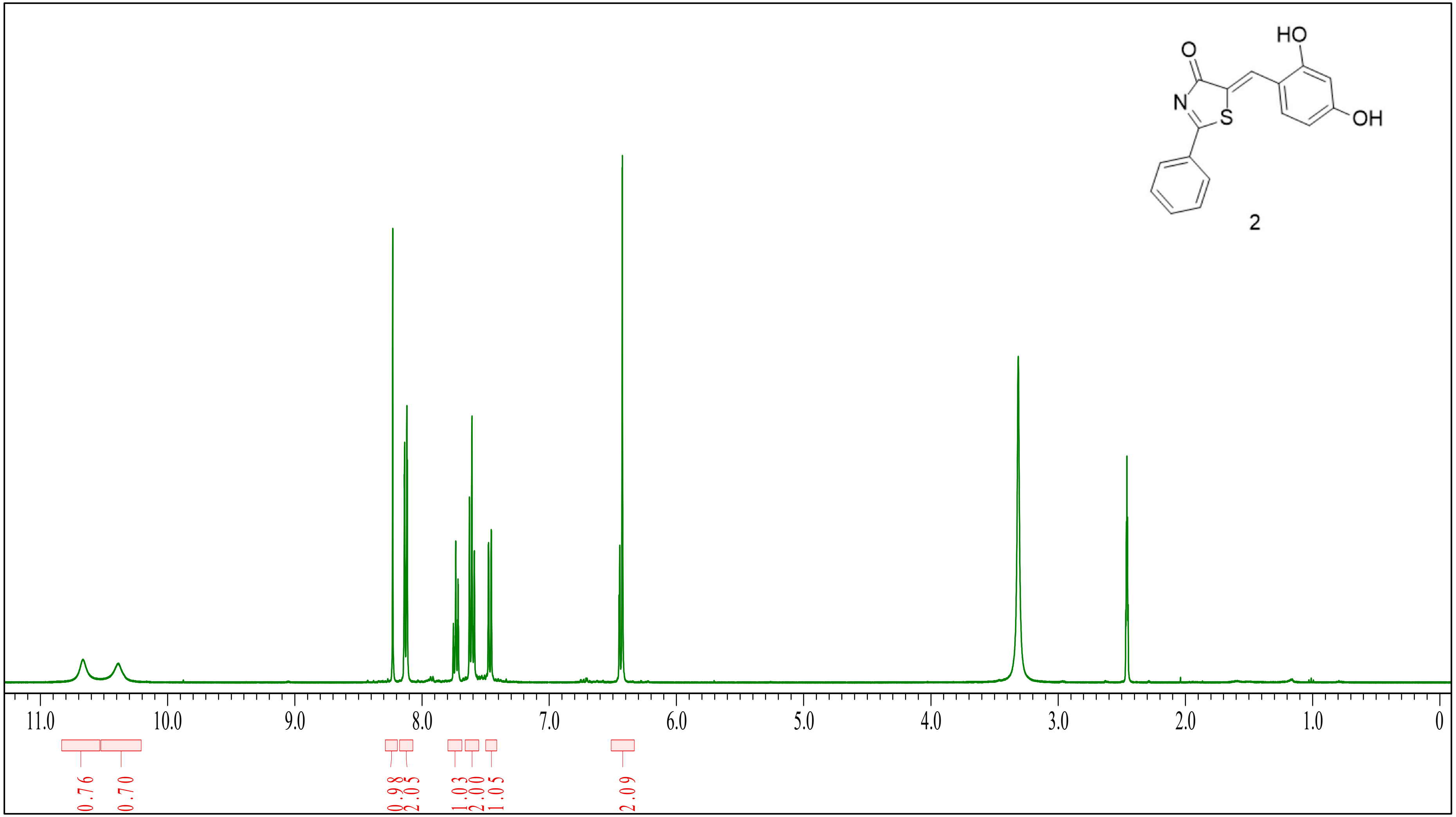

Figure S4. ^{1}H NMR spectrum of compound **2**.

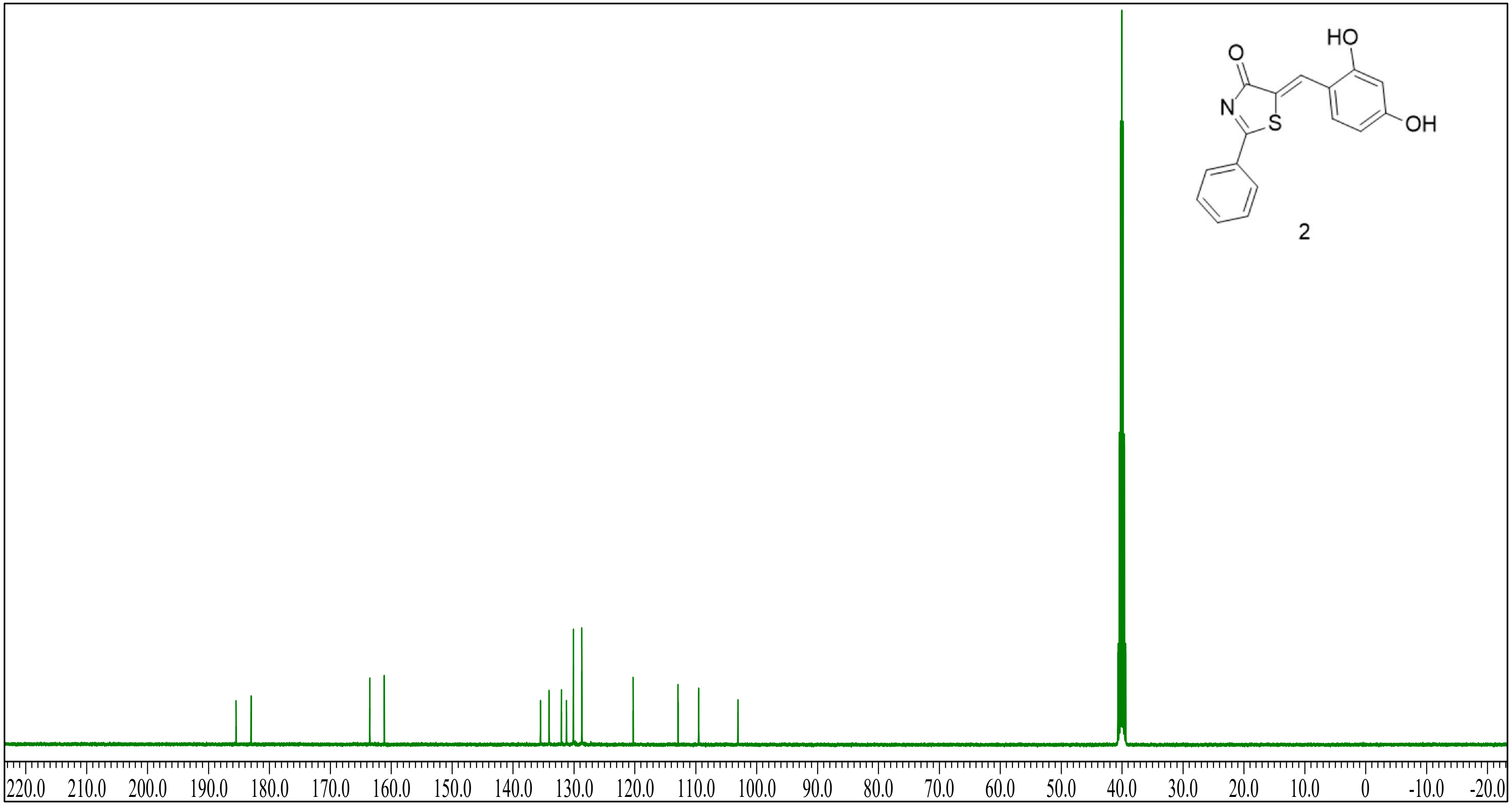

Figure S5. ^{13}C NMR spectrum of compound **2**.

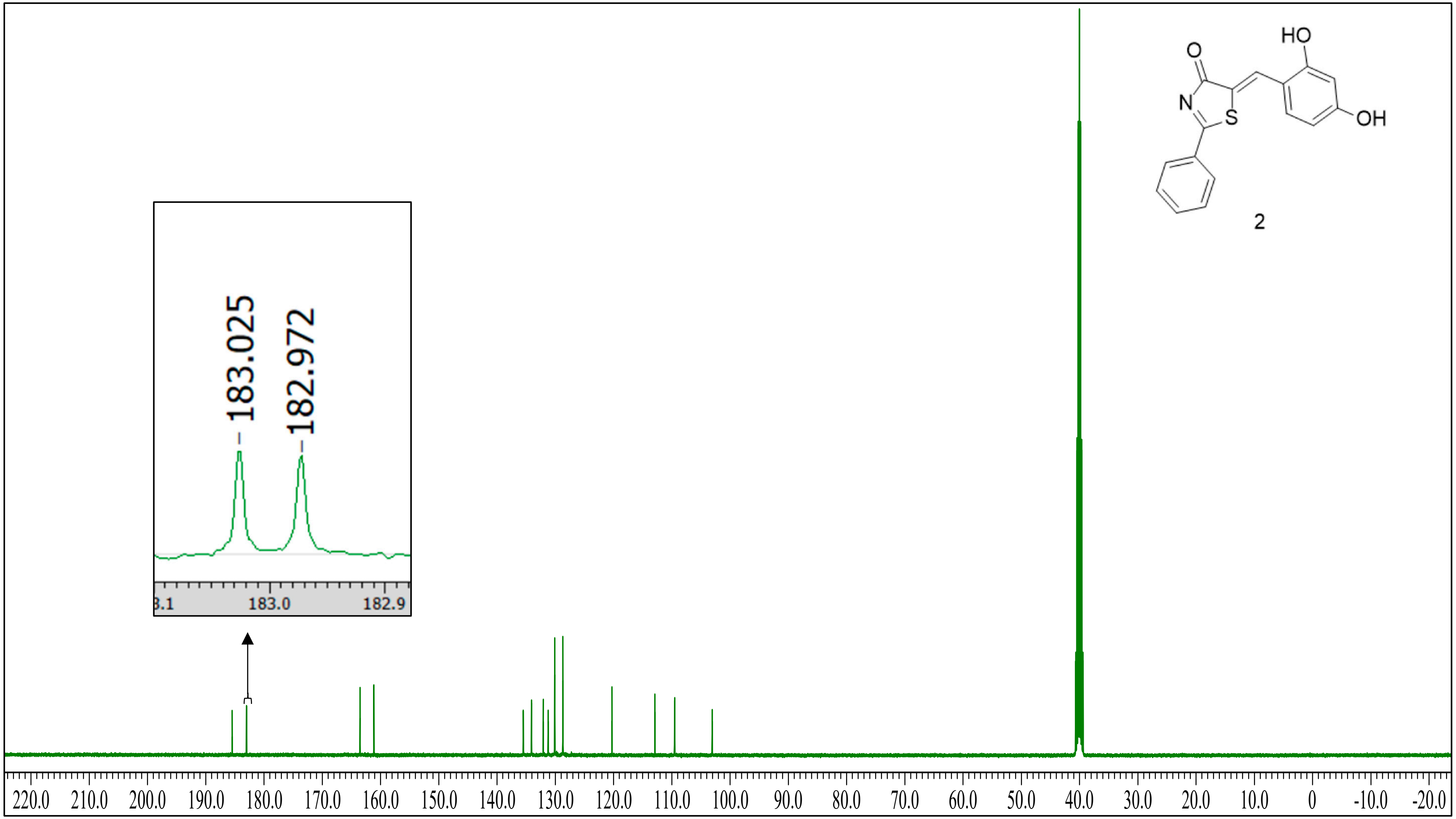

Figure S6. Proton-coupled ^{13}C NMR spectrum of compound **2**.

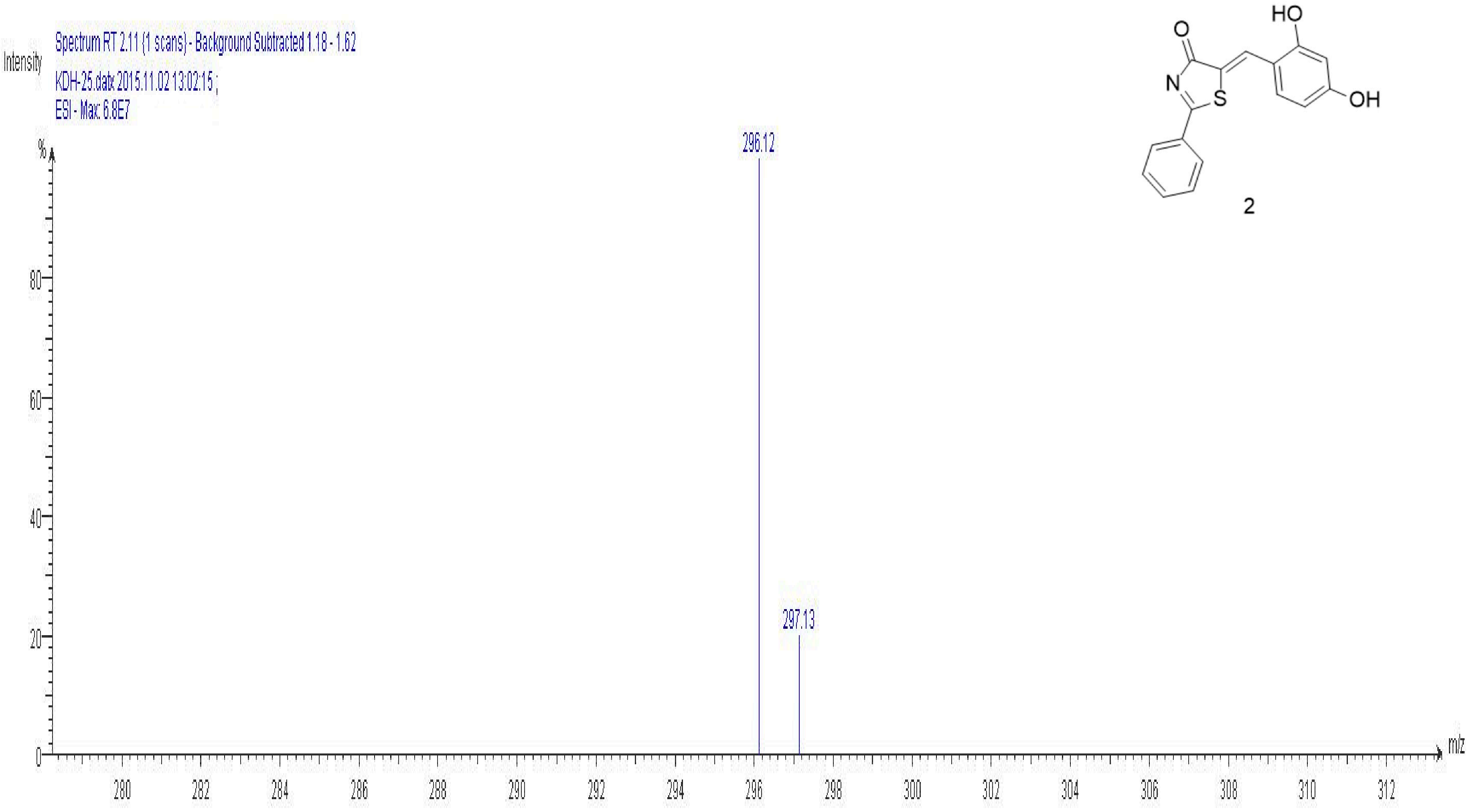

2

Figure S7. LRMS spectrum of compound **2**.

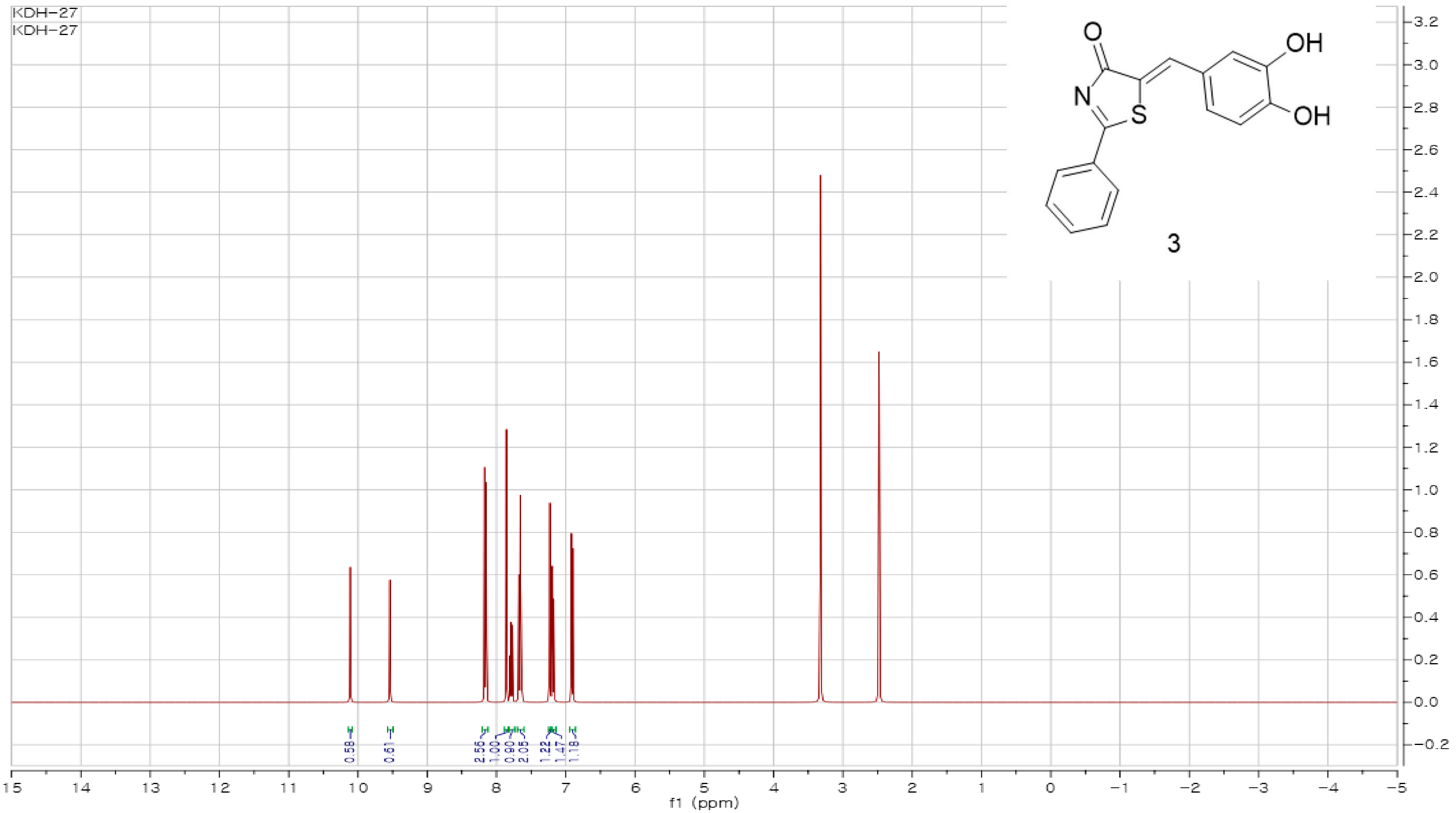

Figure S8. ^{1}H NMR spectrum of compound **3**.

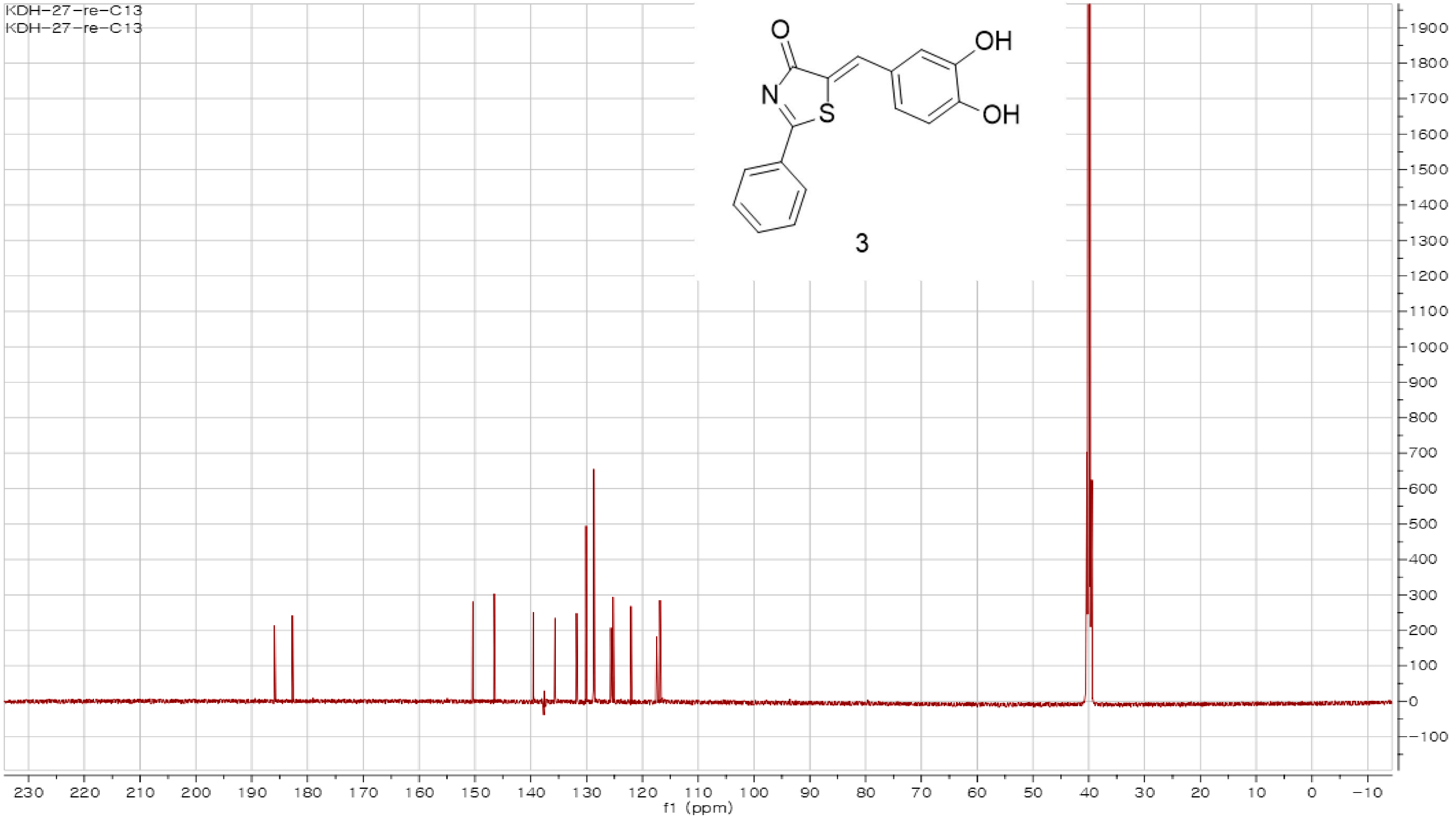

Figure S9. ^{13}C NMR spectrum of compound **3**.

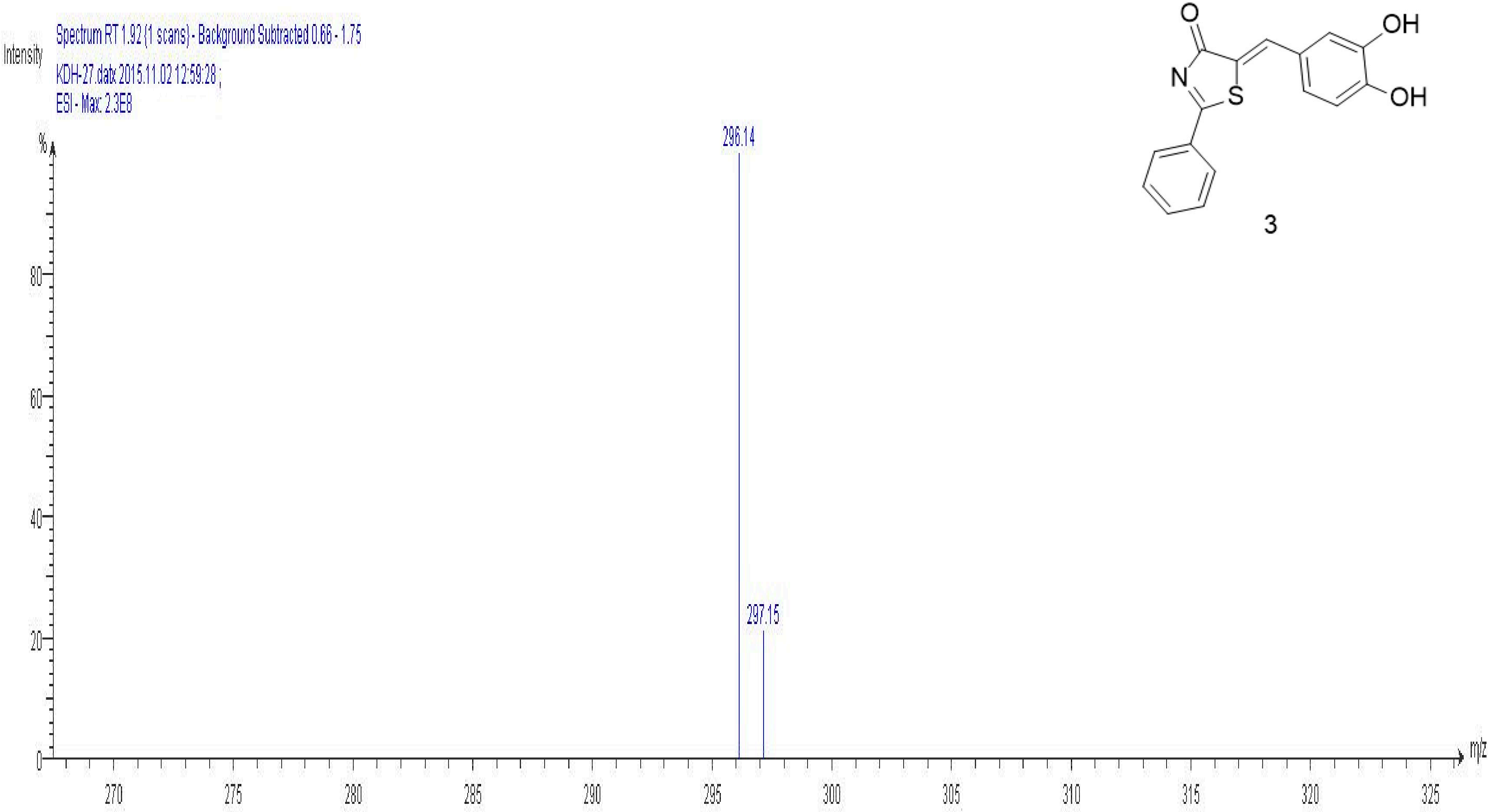

3

Figure S10. LRMS spectrum of compound **3**.

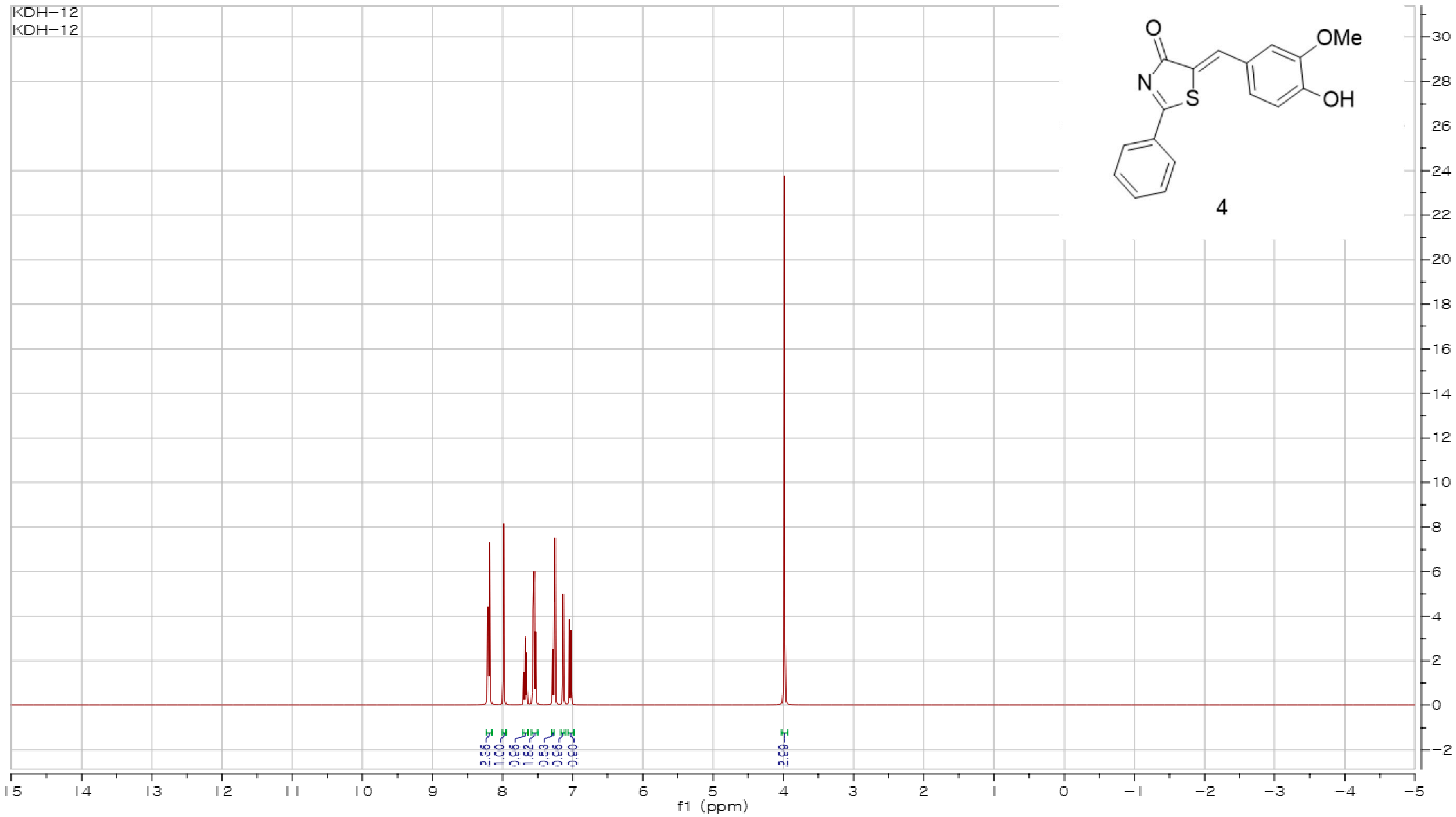

Figure S11. ^{1}H NMR spectrum of compound **4**.

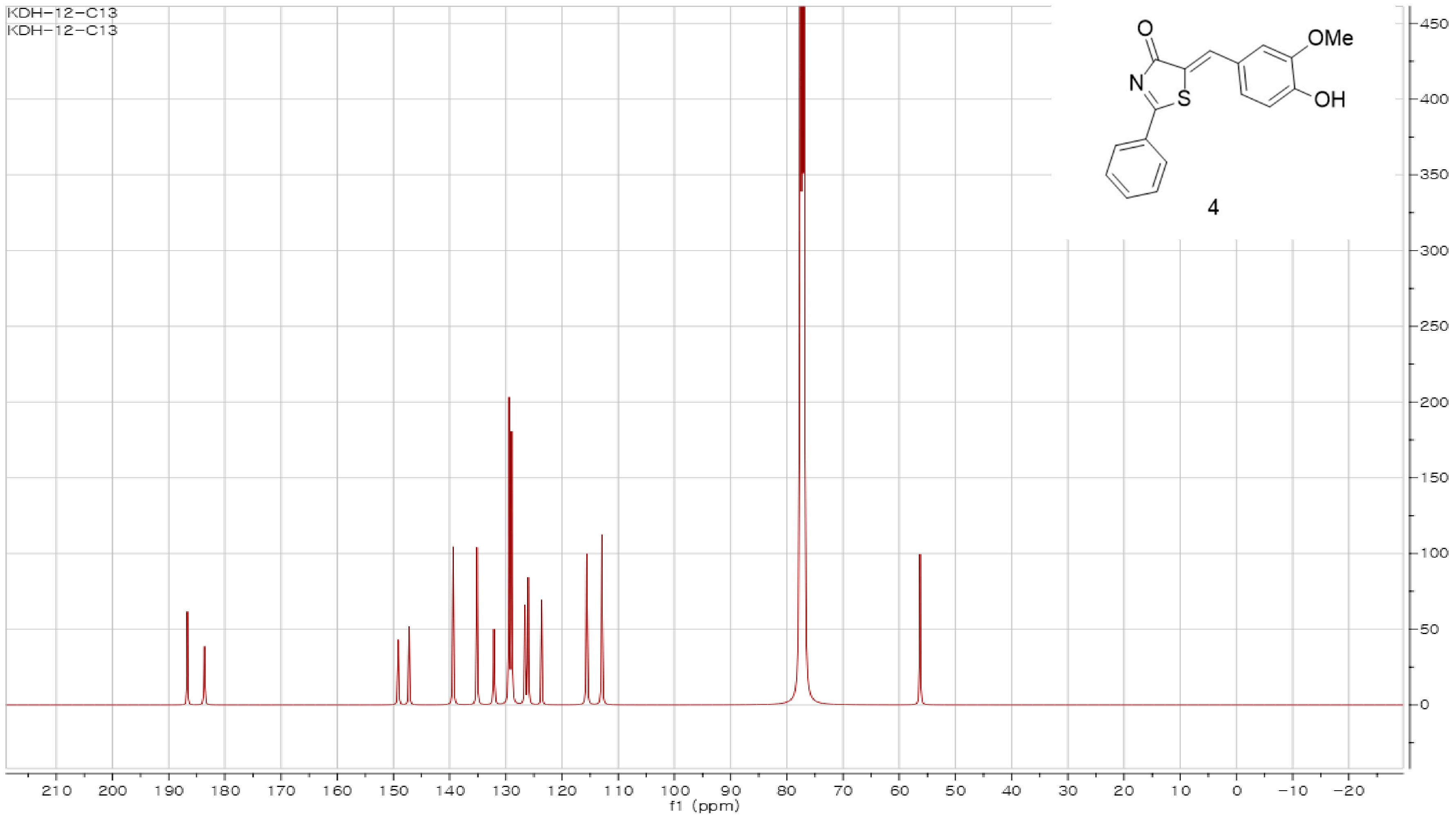

Figure S12. ^{13}C NMR spectrum of compound **4**.

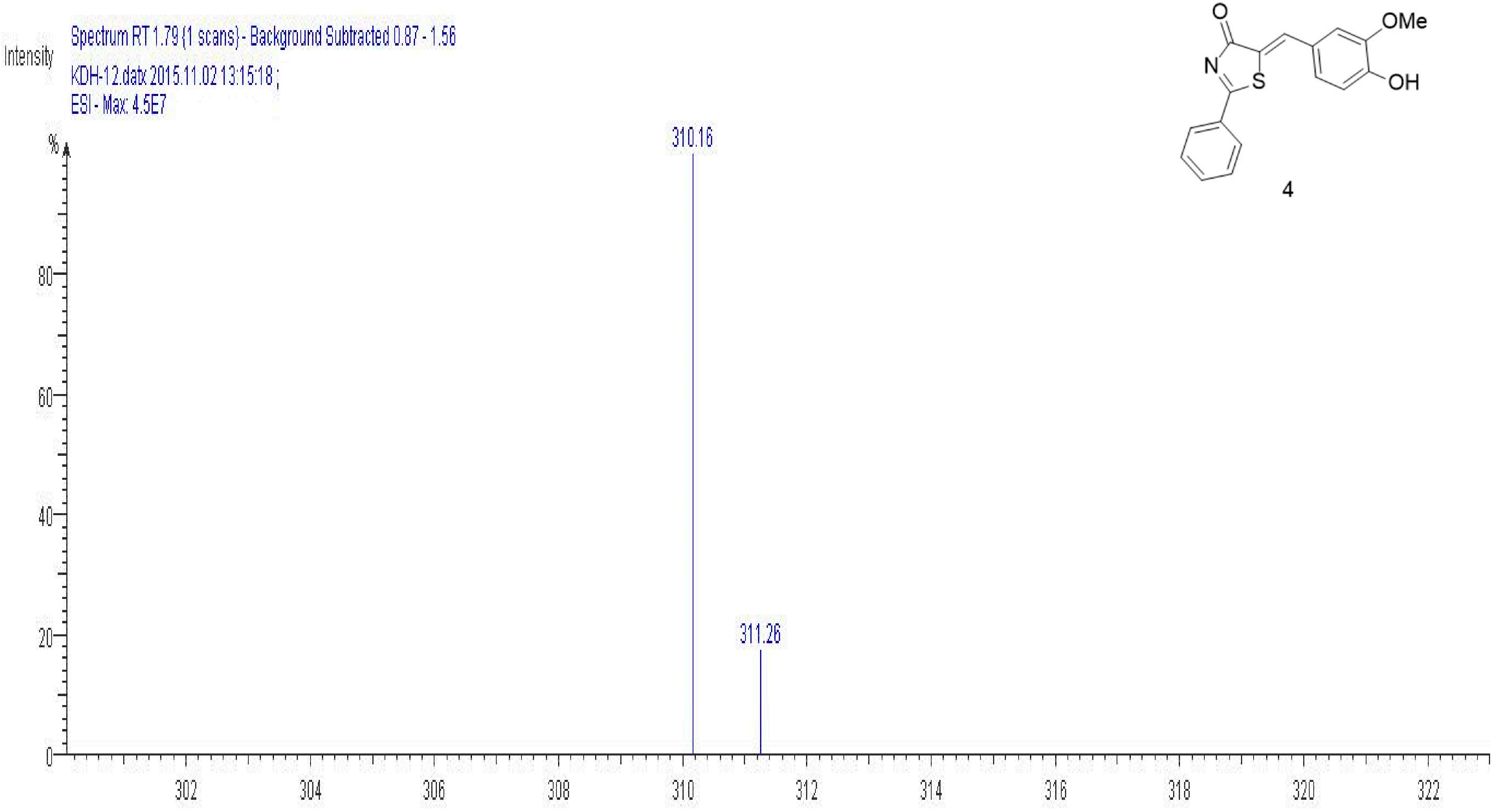

4

Figure S13. LRMS spectrum of compound **4**.

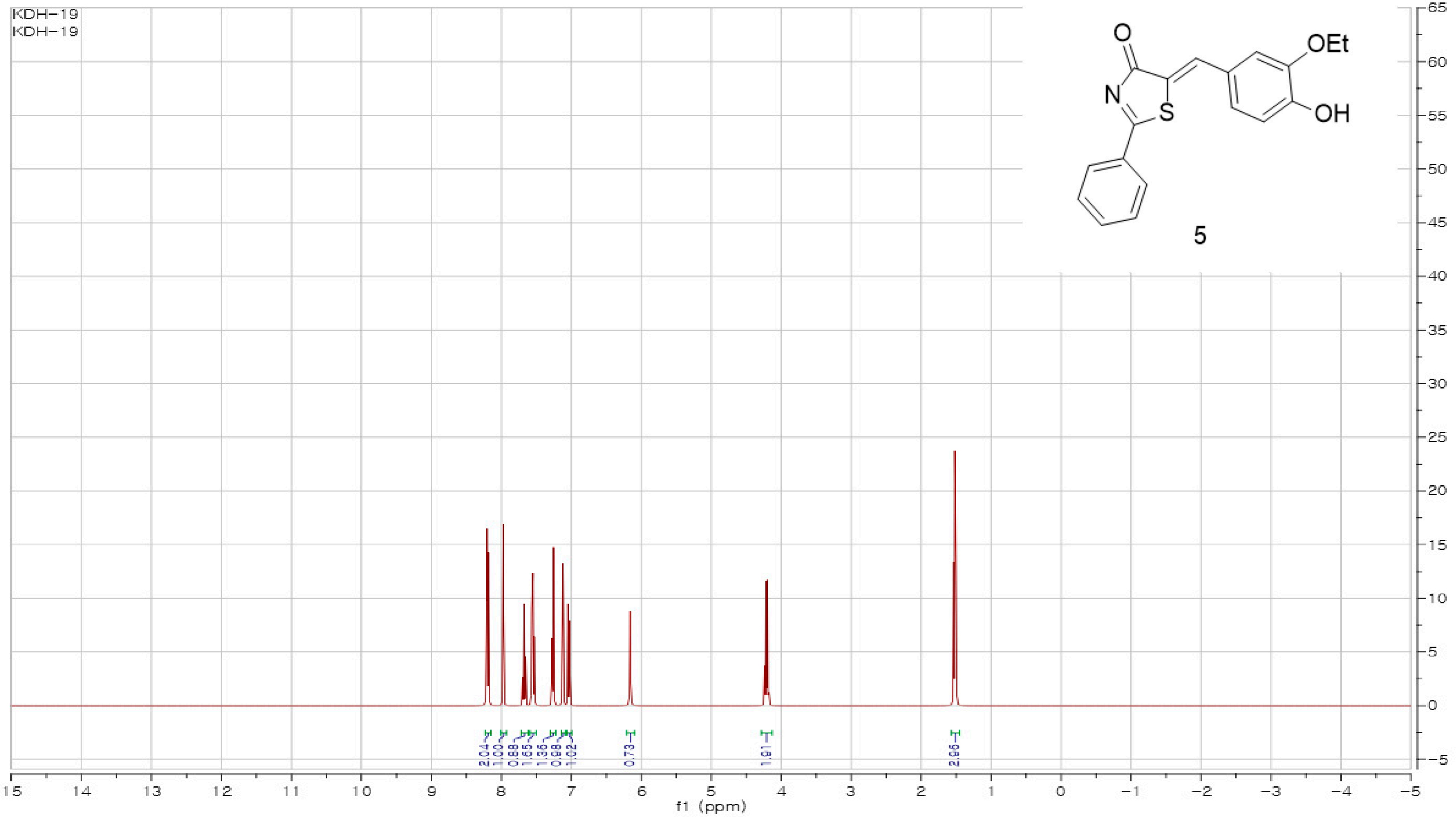

Figure S14. ^{1}H NMR spectrum of compound **5**.

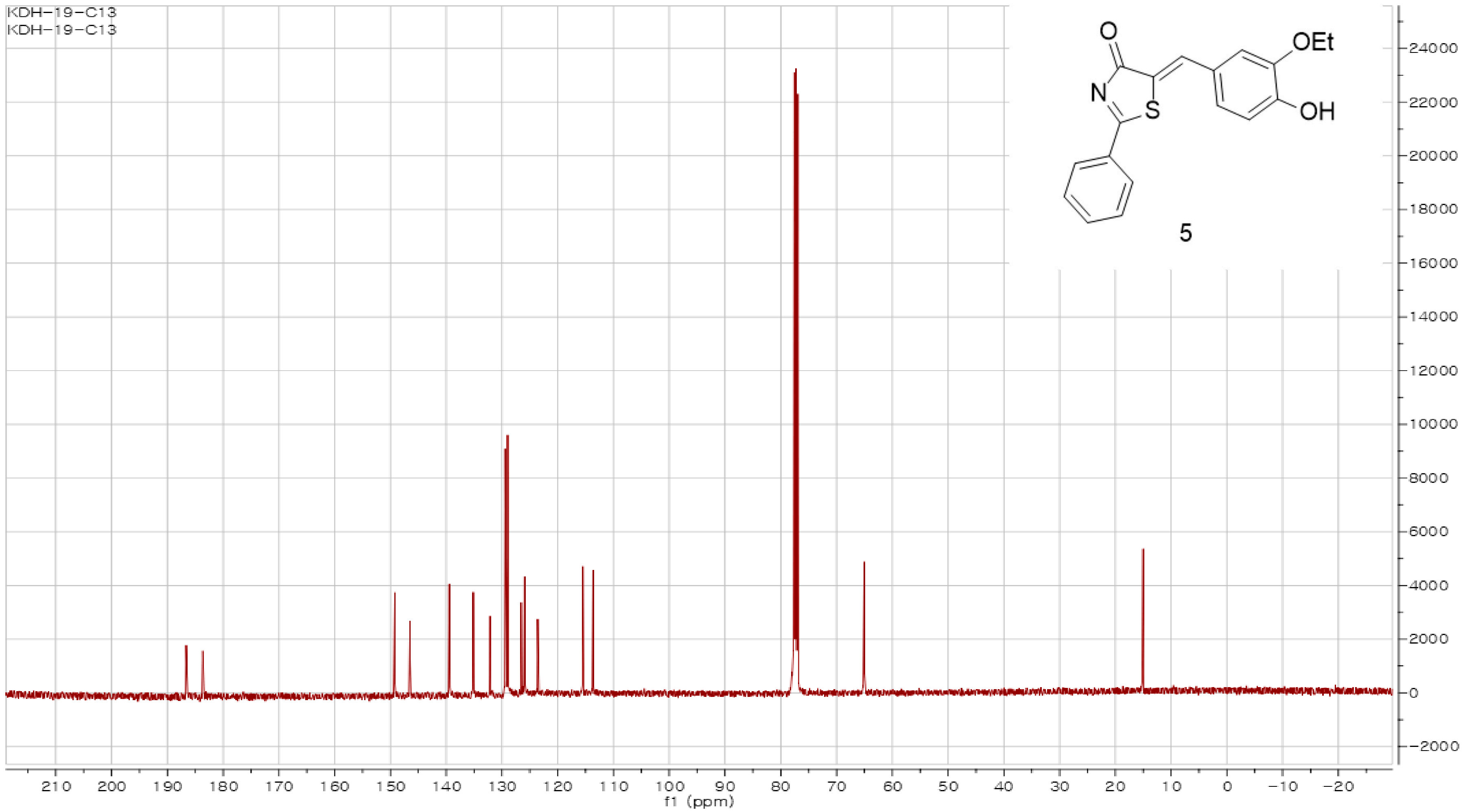

Figure S15. ^{13}C NMR spectrum of compound **5**.

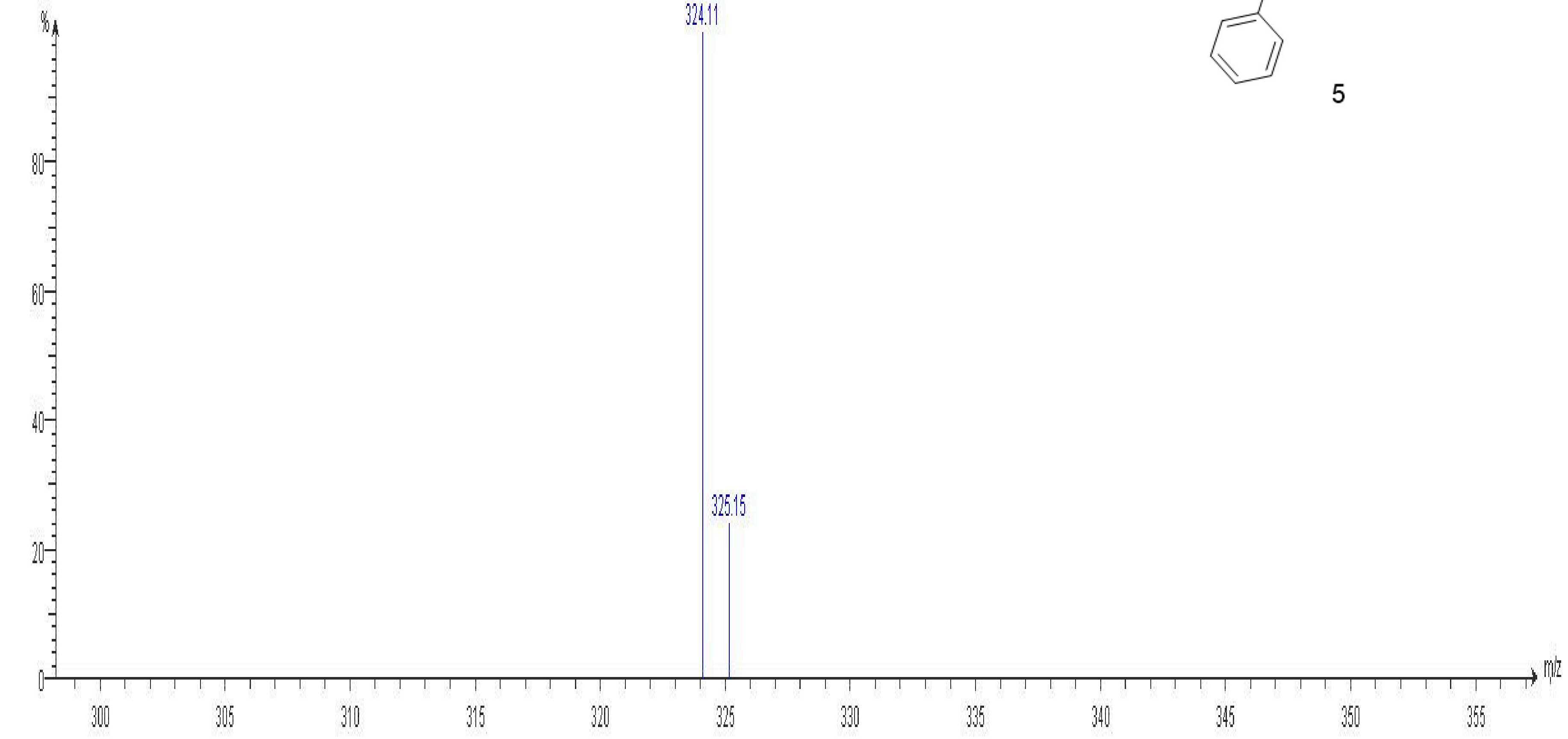

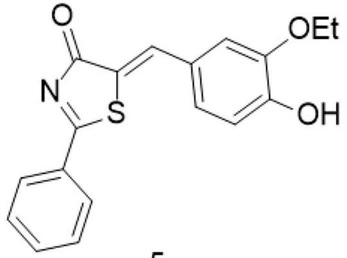

5

Figure S16. LRMS spectrum of compound **5**.

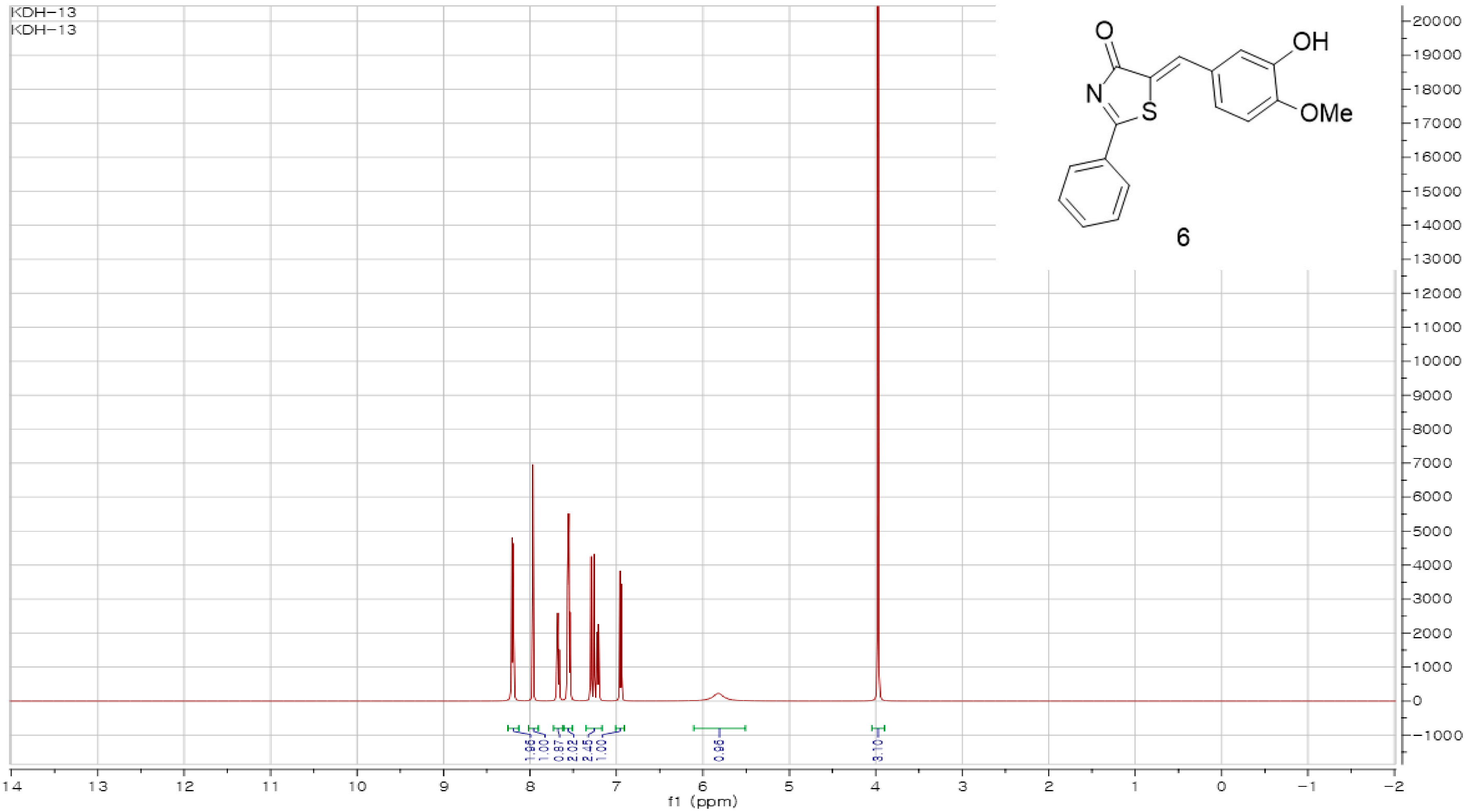

Figure S17. ^{1}H NMR spectrum of compound **6**.

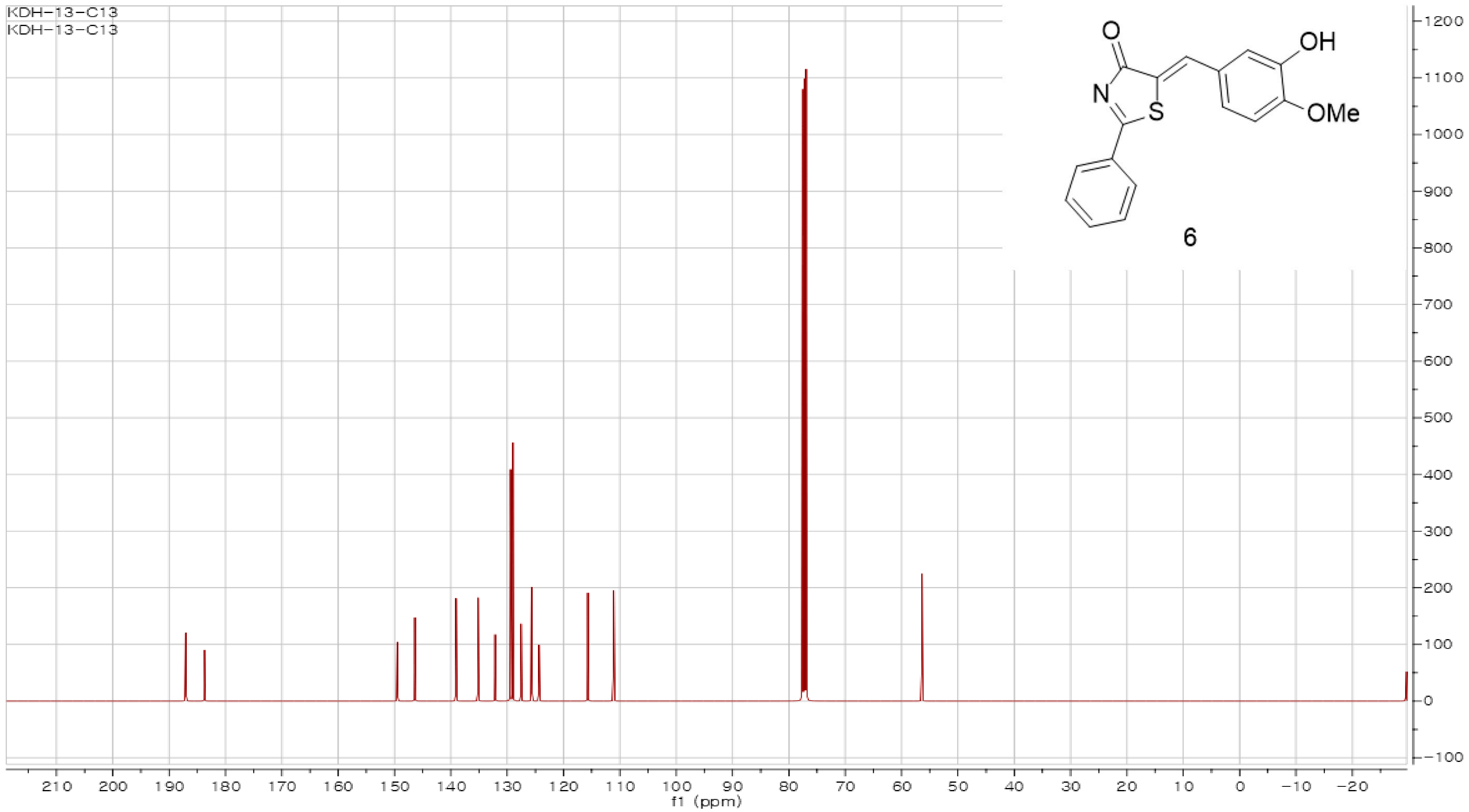

Figure S18. ^{13}C NMR spectrum of compound **6**.

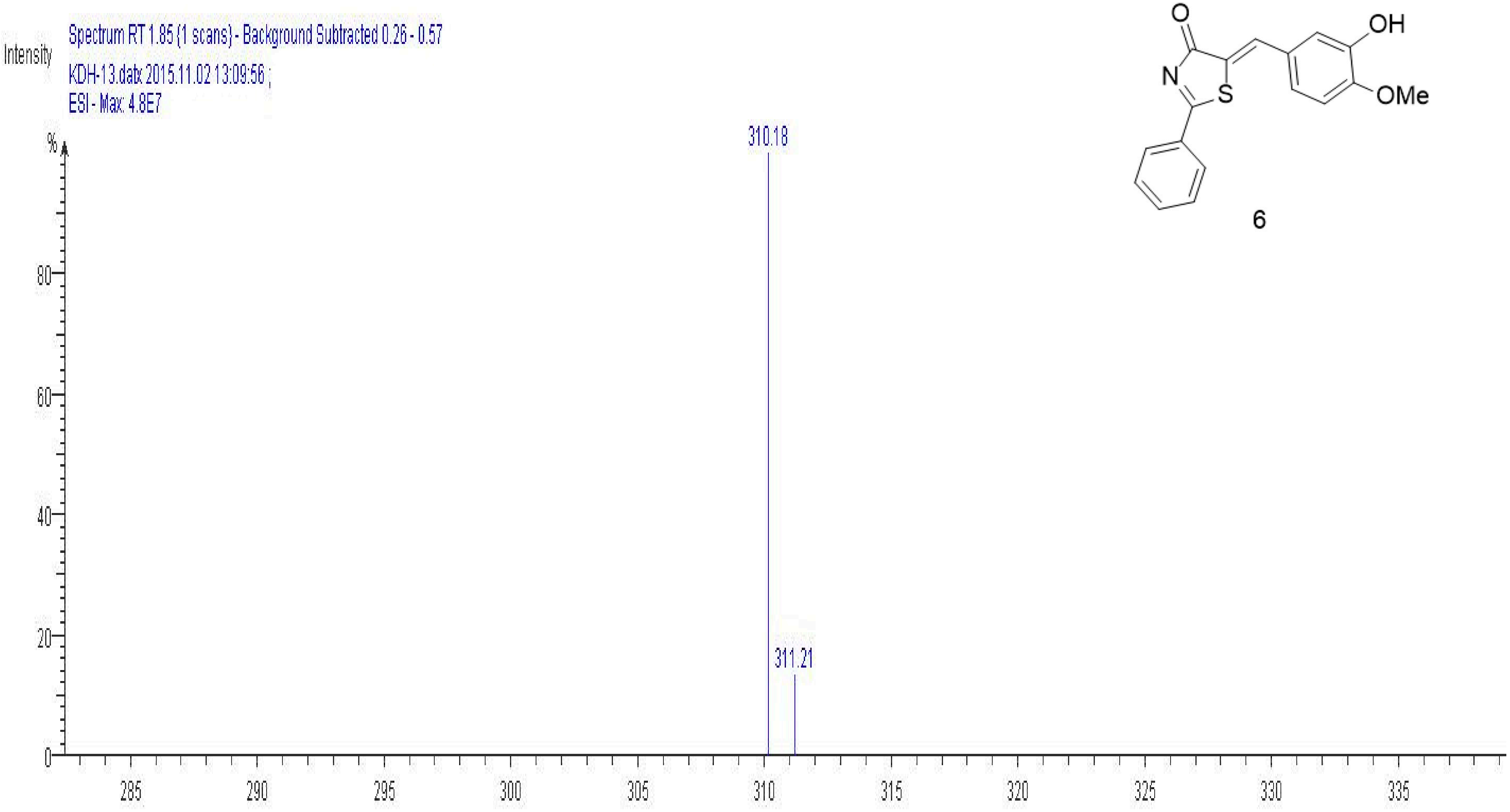

Figure S19. LRMS spectrum of compound **6**.

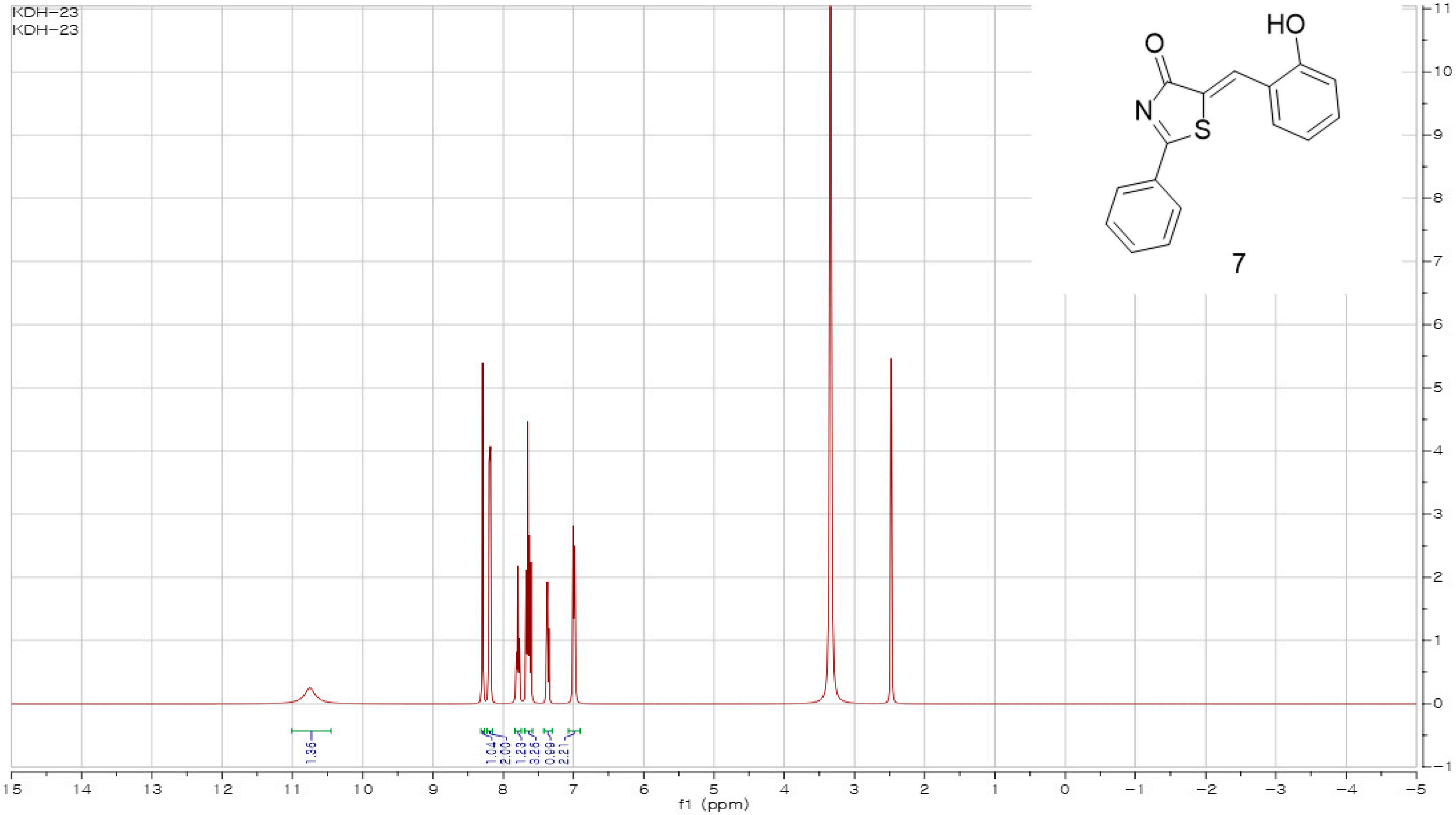

Figure S20. ^{1}H NMR spectrum of compound **7**.

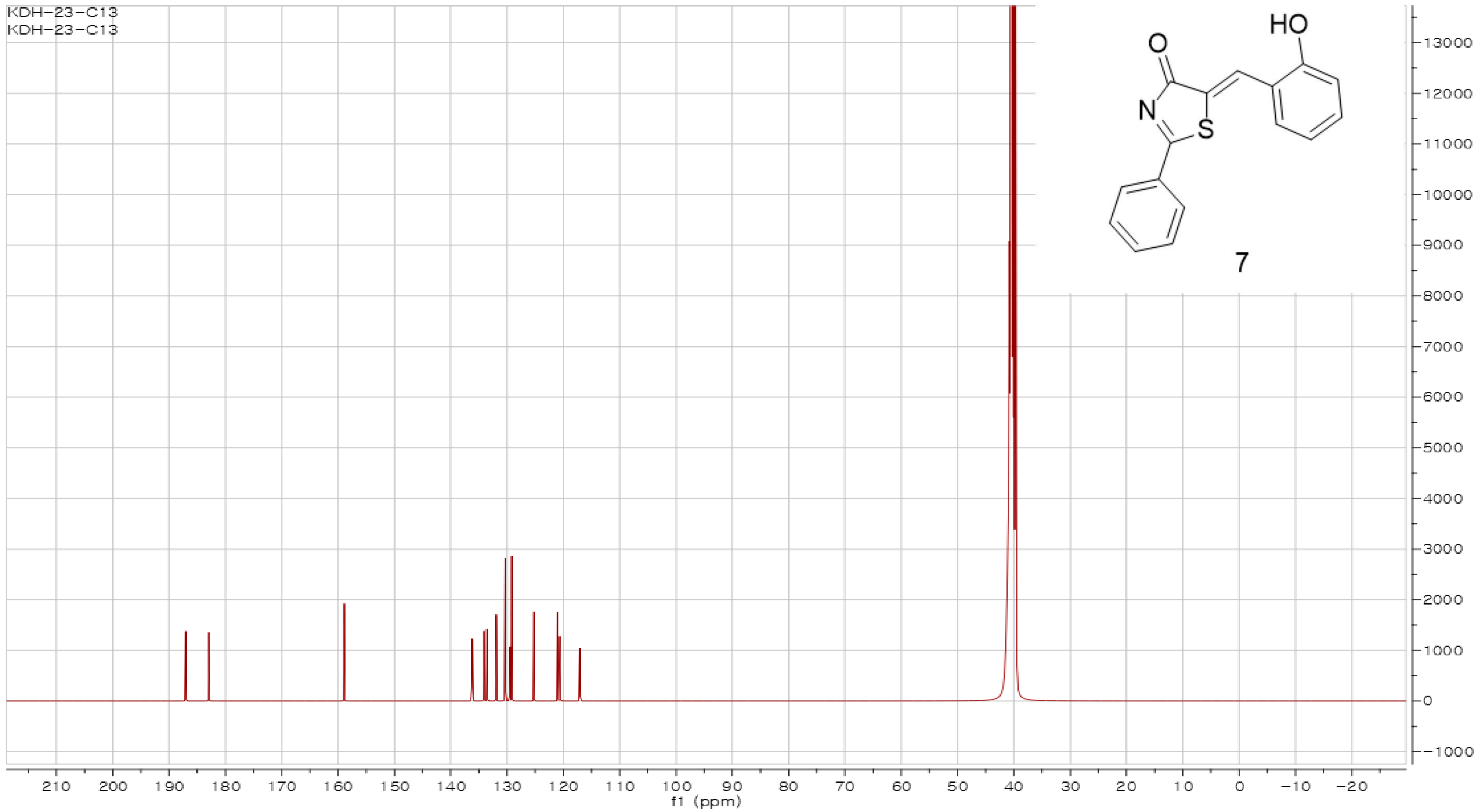

Figure S21. ^{13}C NMR spectrum of compound **7**.

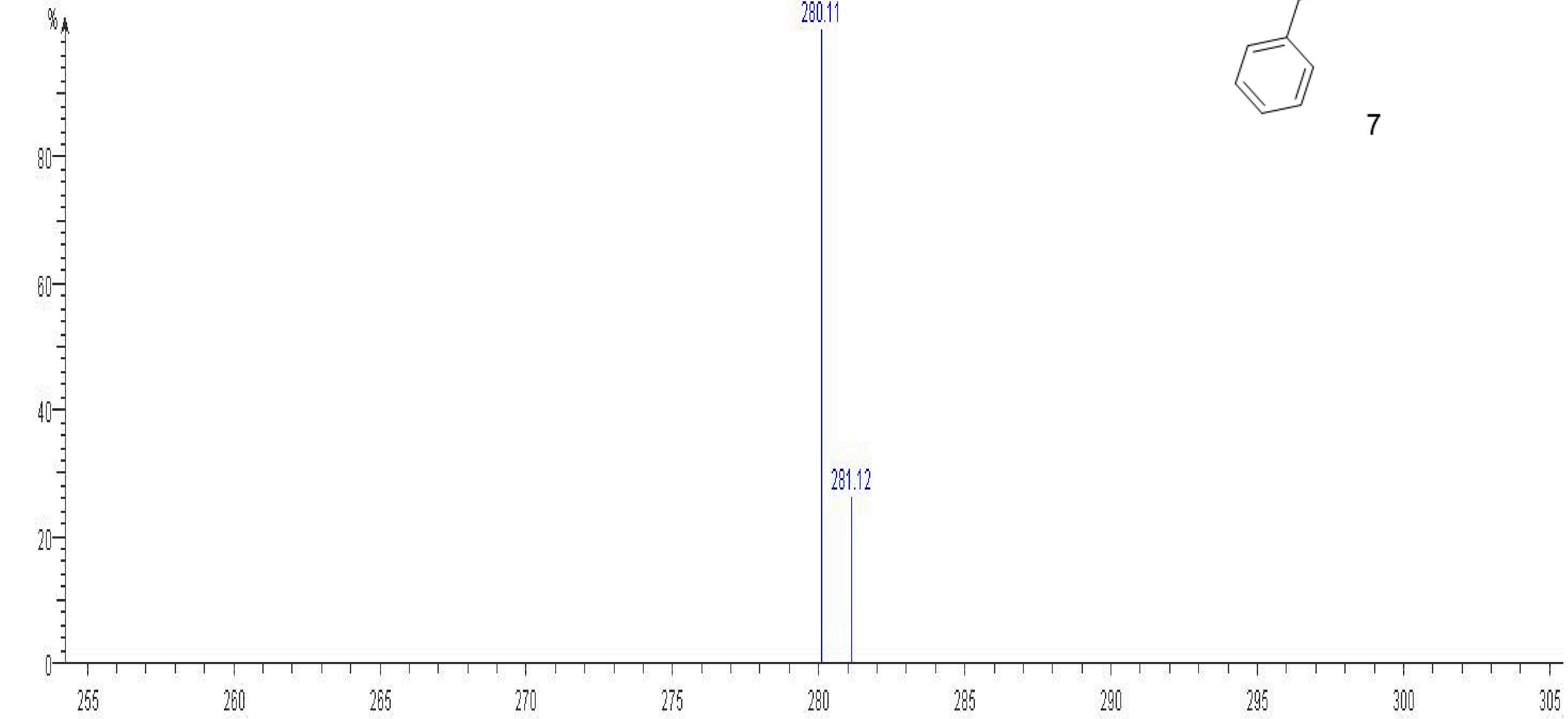

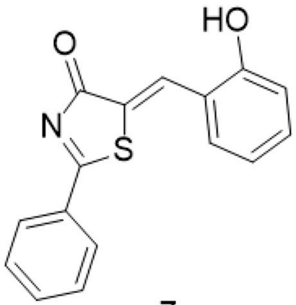

7

Figure S22. LRMS spectrum of compound **7**.

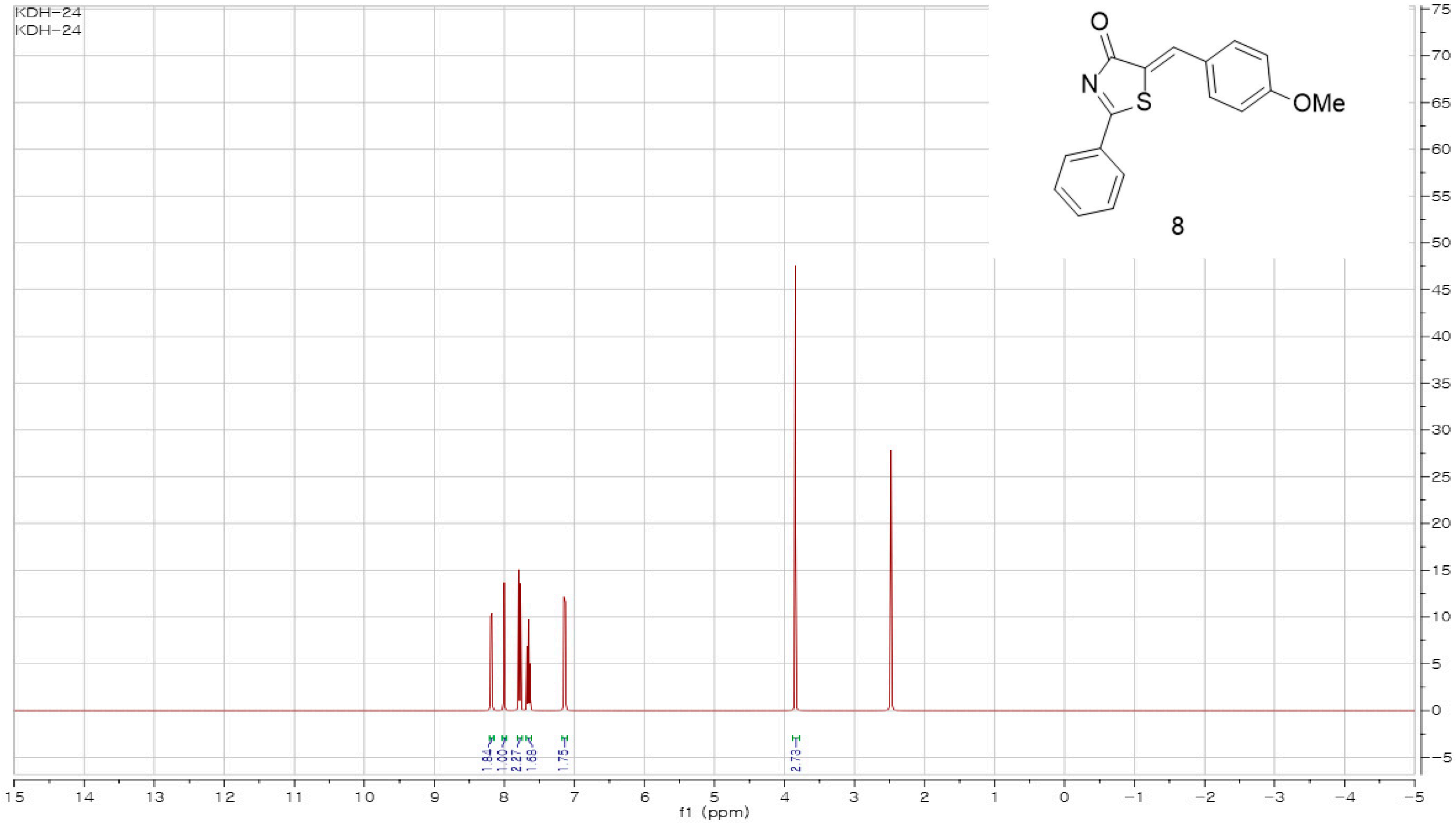

Figure S23. ^{1}H NMR spectrum of compound **8**.

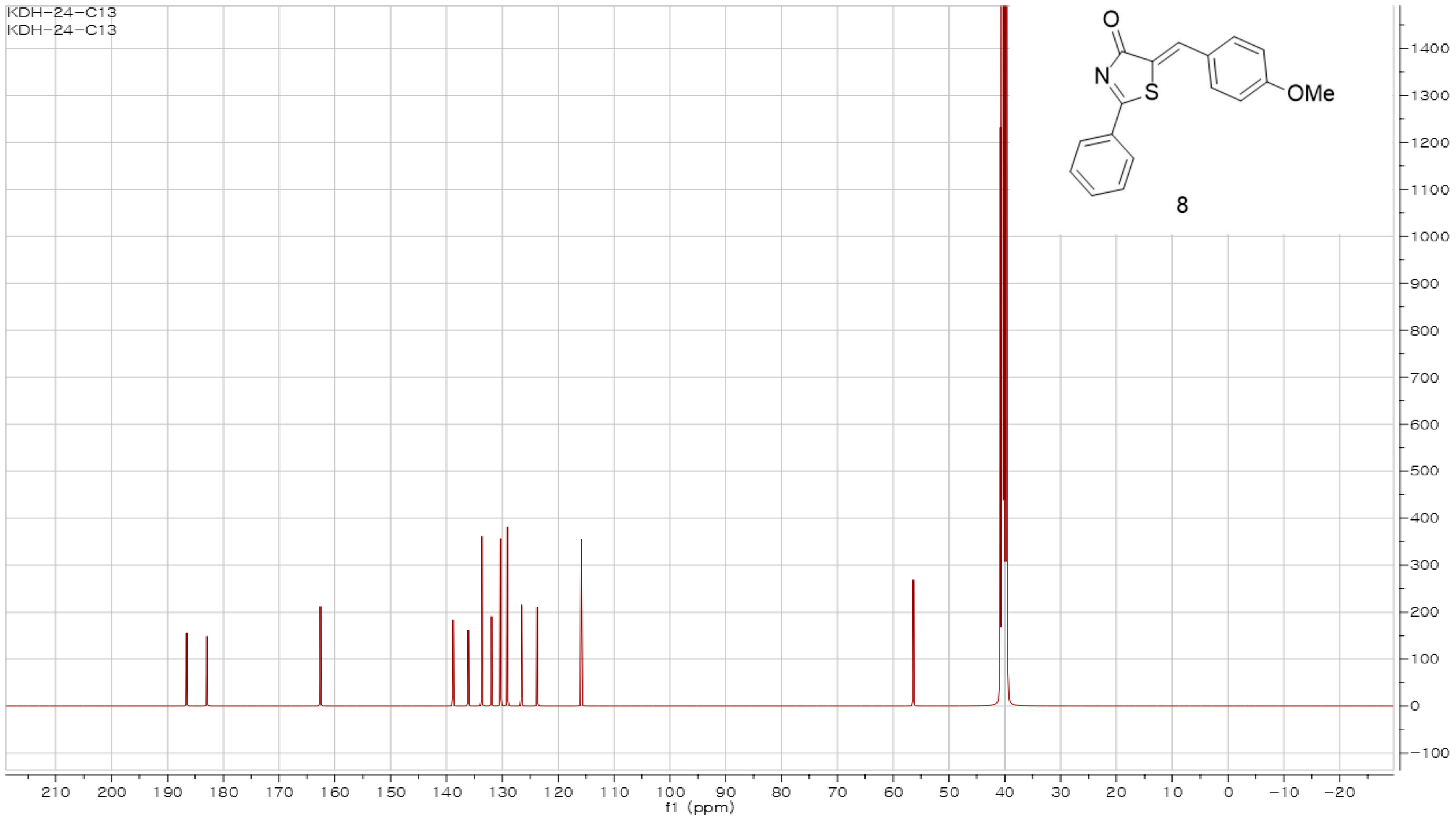

Figure S24. ^{13}C NMR spectrum of compound **8**.

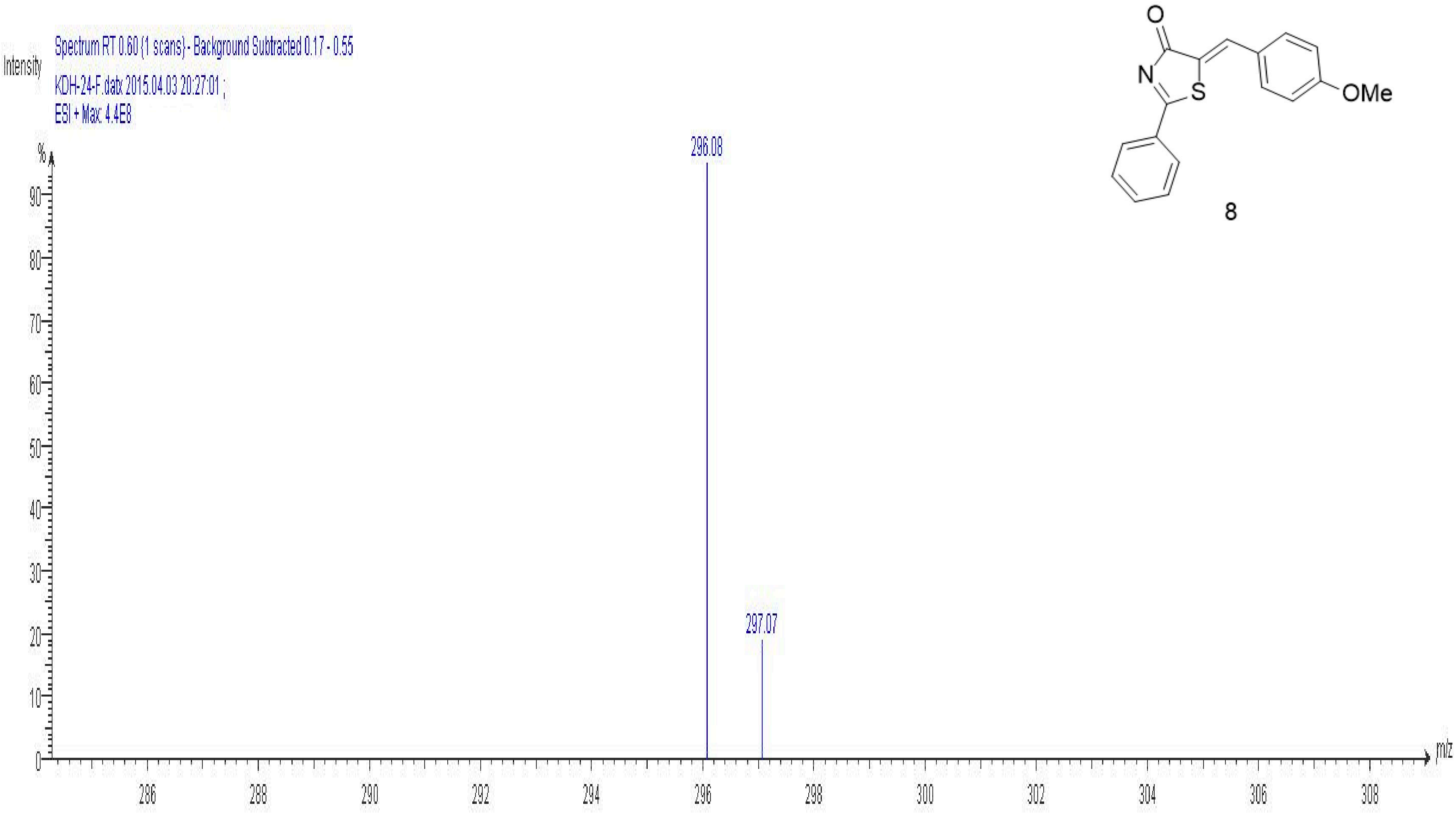

Figure S25. LRMS spectrum of compound **8**.

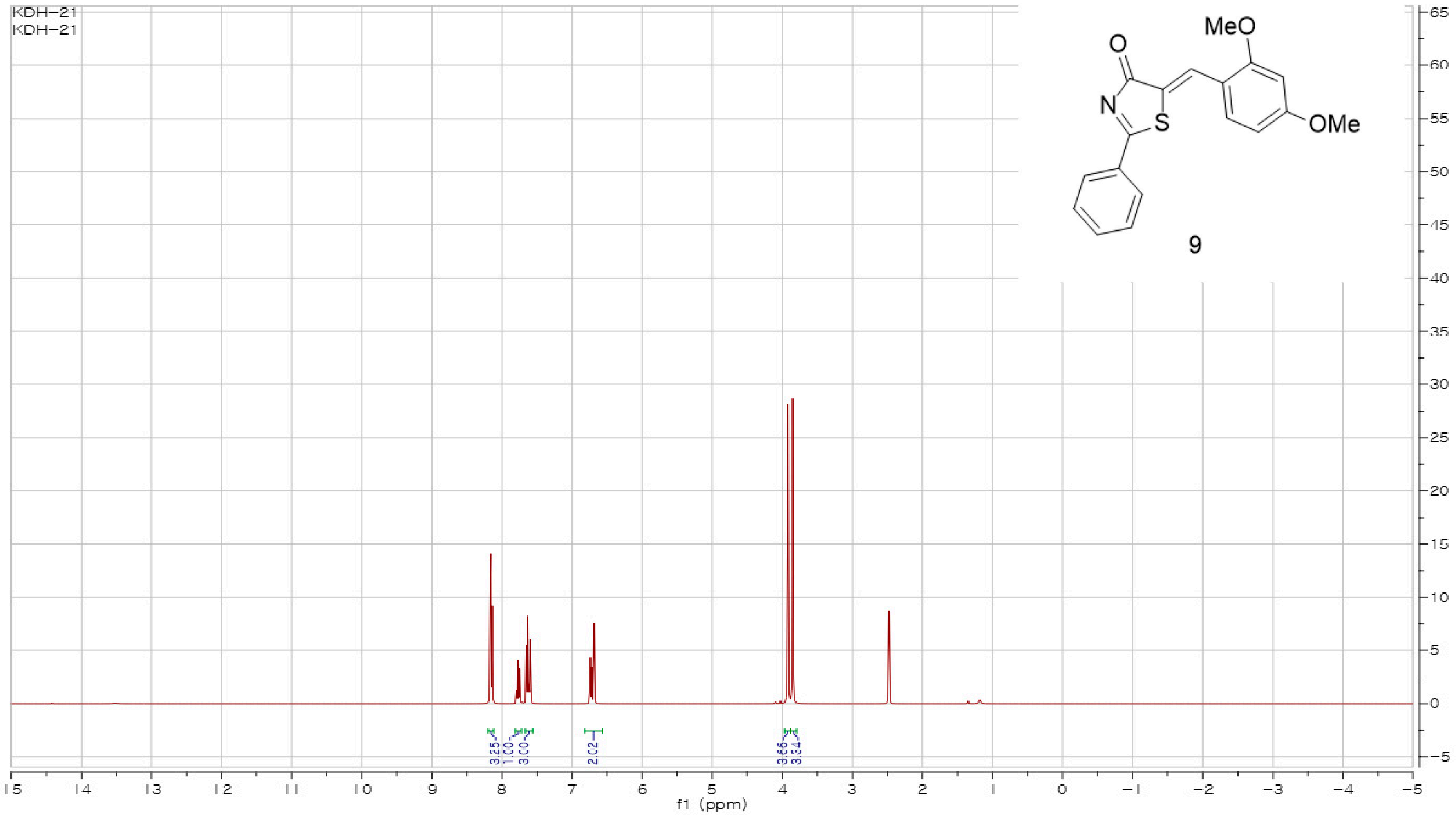

Figure S26. ^{1}H NMR spectrum of compound **9**.

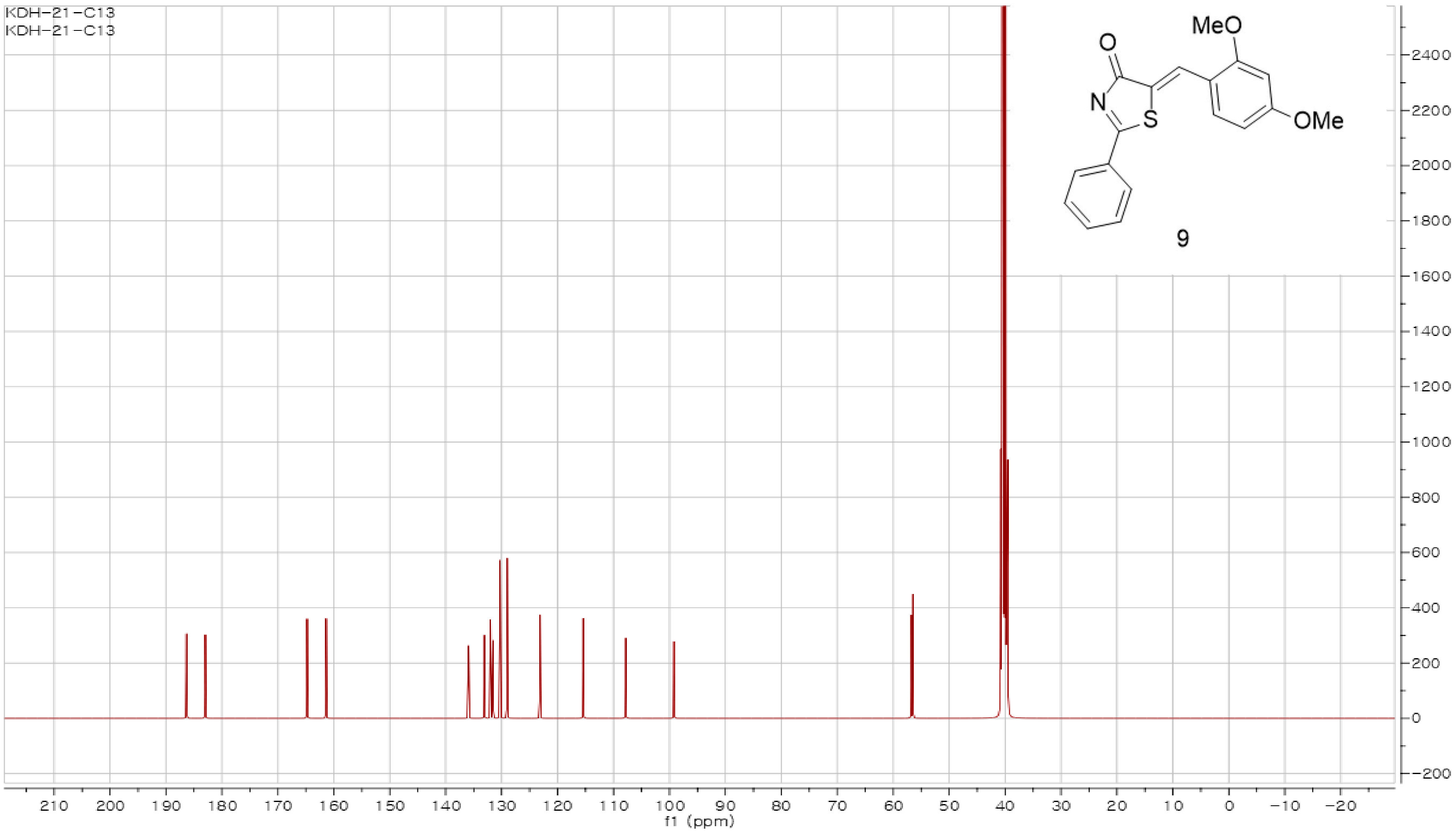

Figure S27. ^{13}C NMR spectrum of compound **9**.

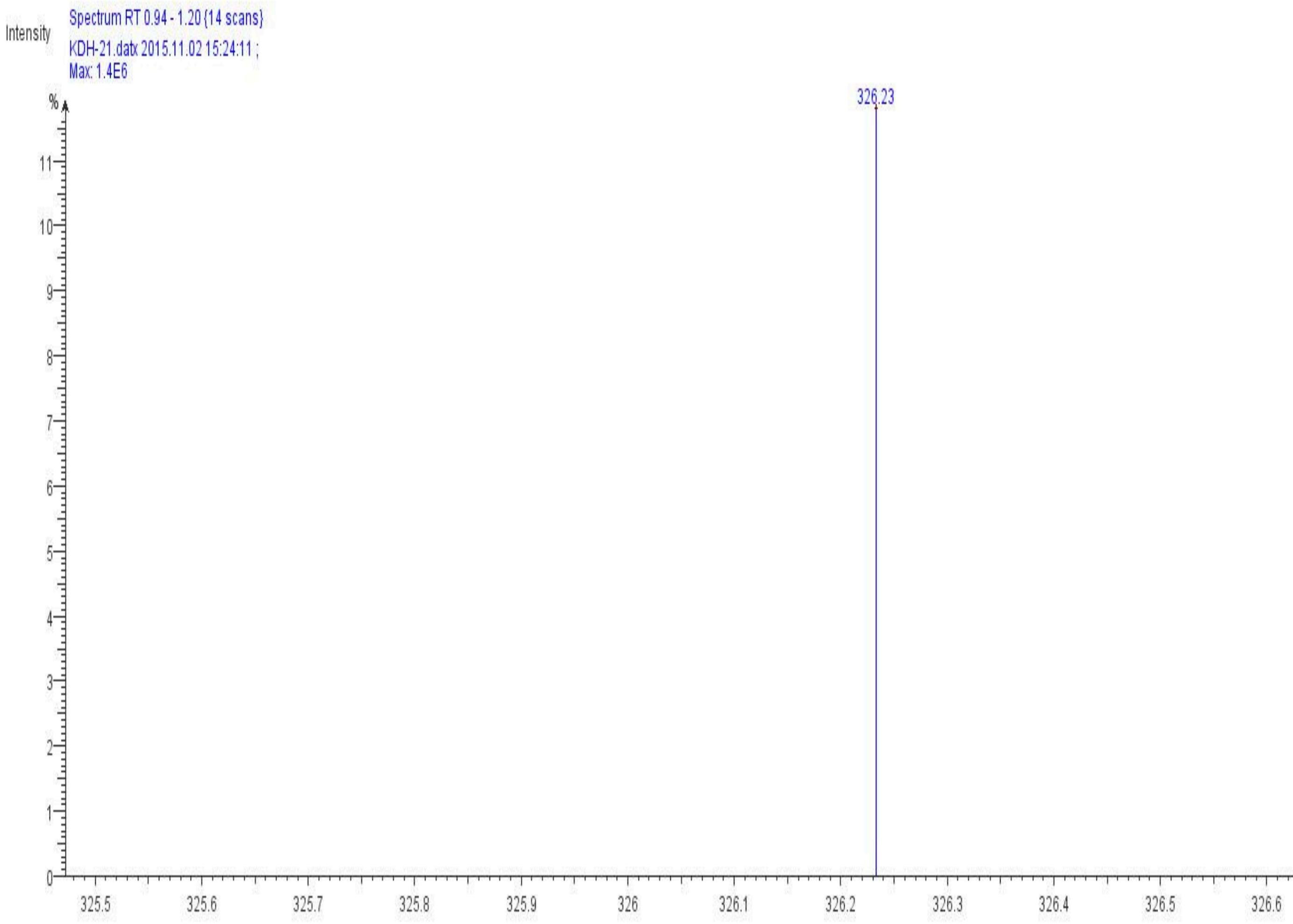

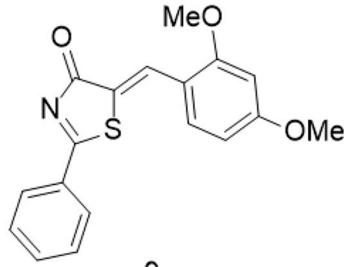

9

Figure S28. LRMS spectrum of compound **9**.

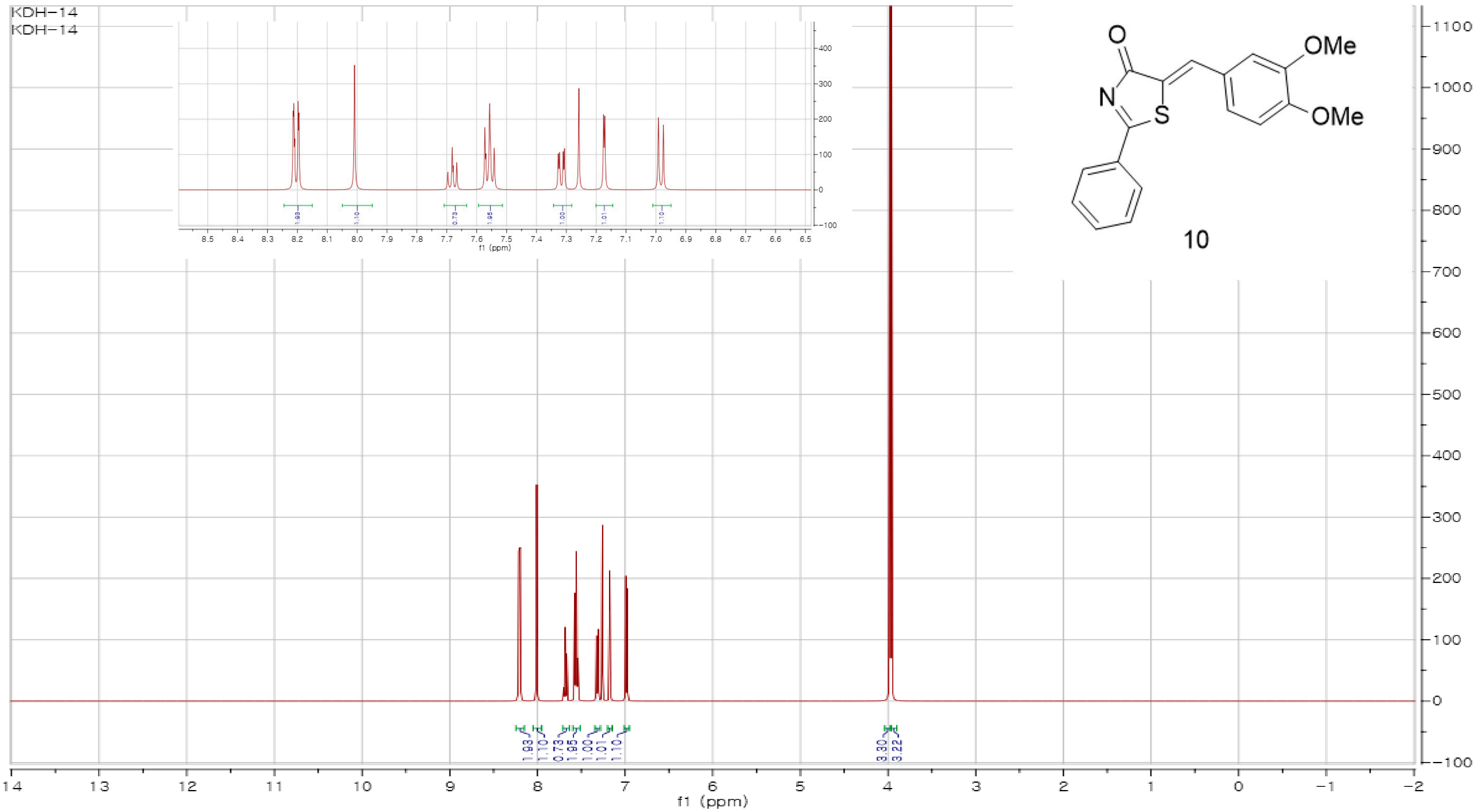

Figure S29. ^{1}H NMR spectrum of compound **10**.

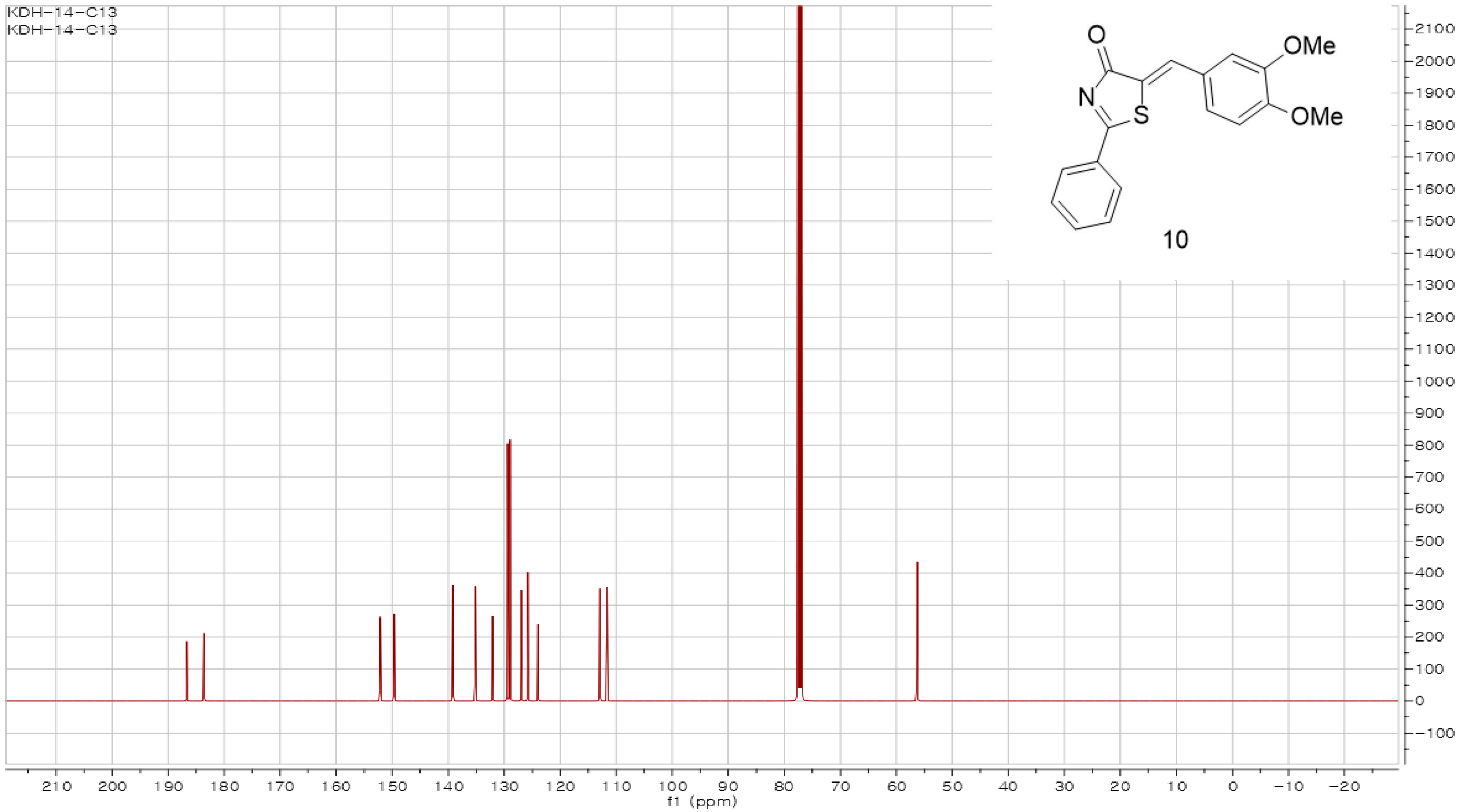

Figure S30. ^{13}C NMR spectrum of compound **10**.

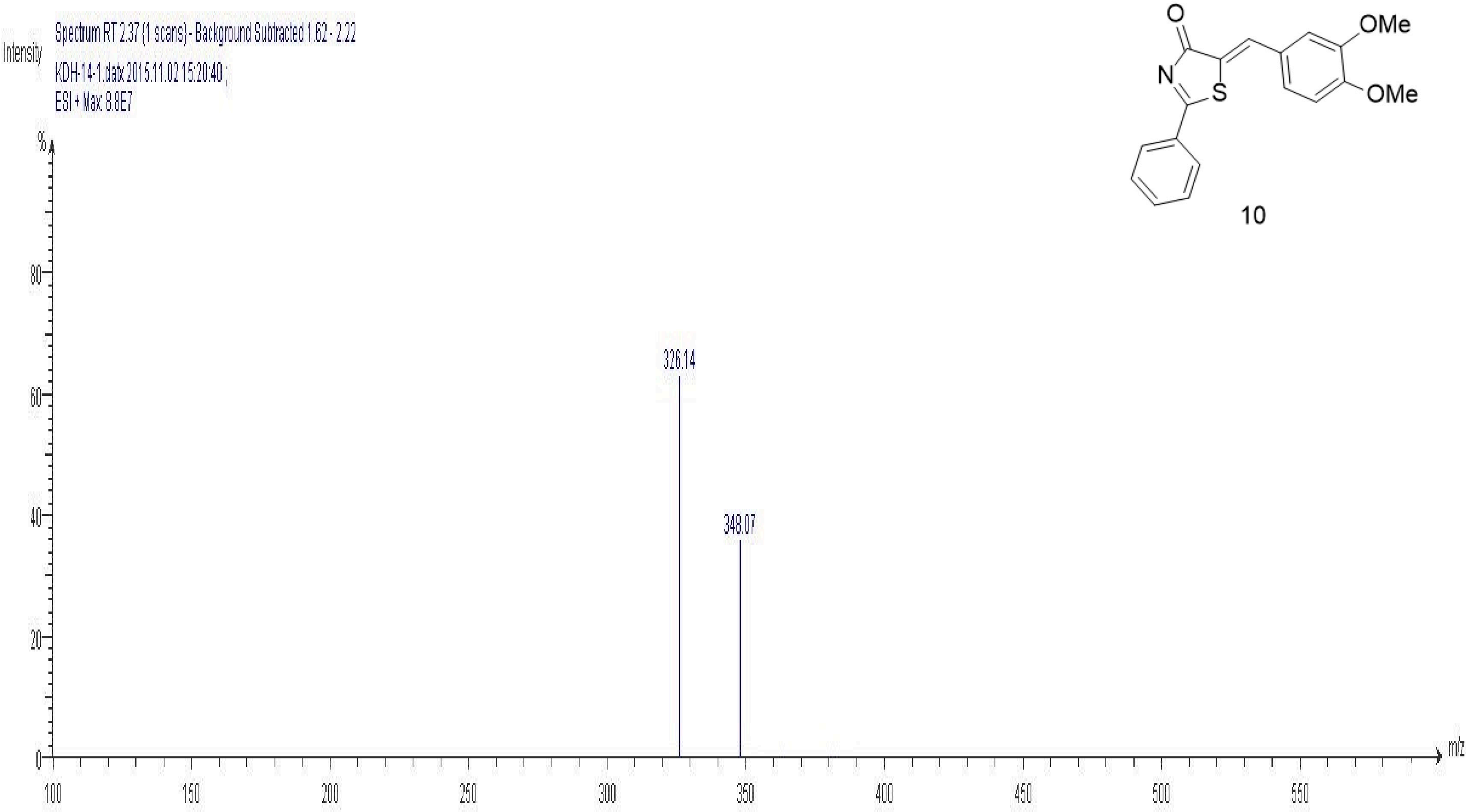

10

Figure S31. LRMS spectrum of compound **10**.

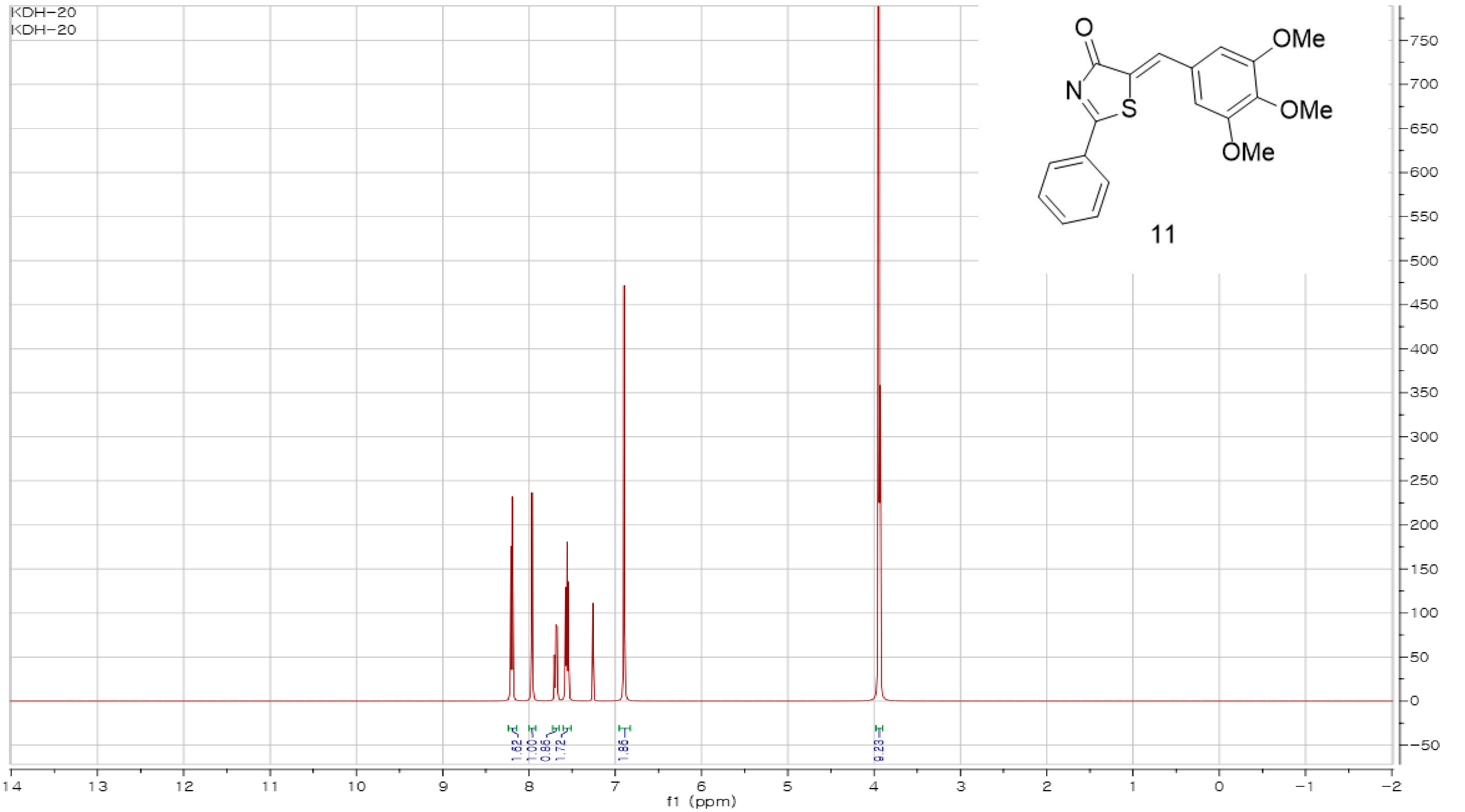

Figure S32. ^{1}H NMR spectrum of compound **11**.

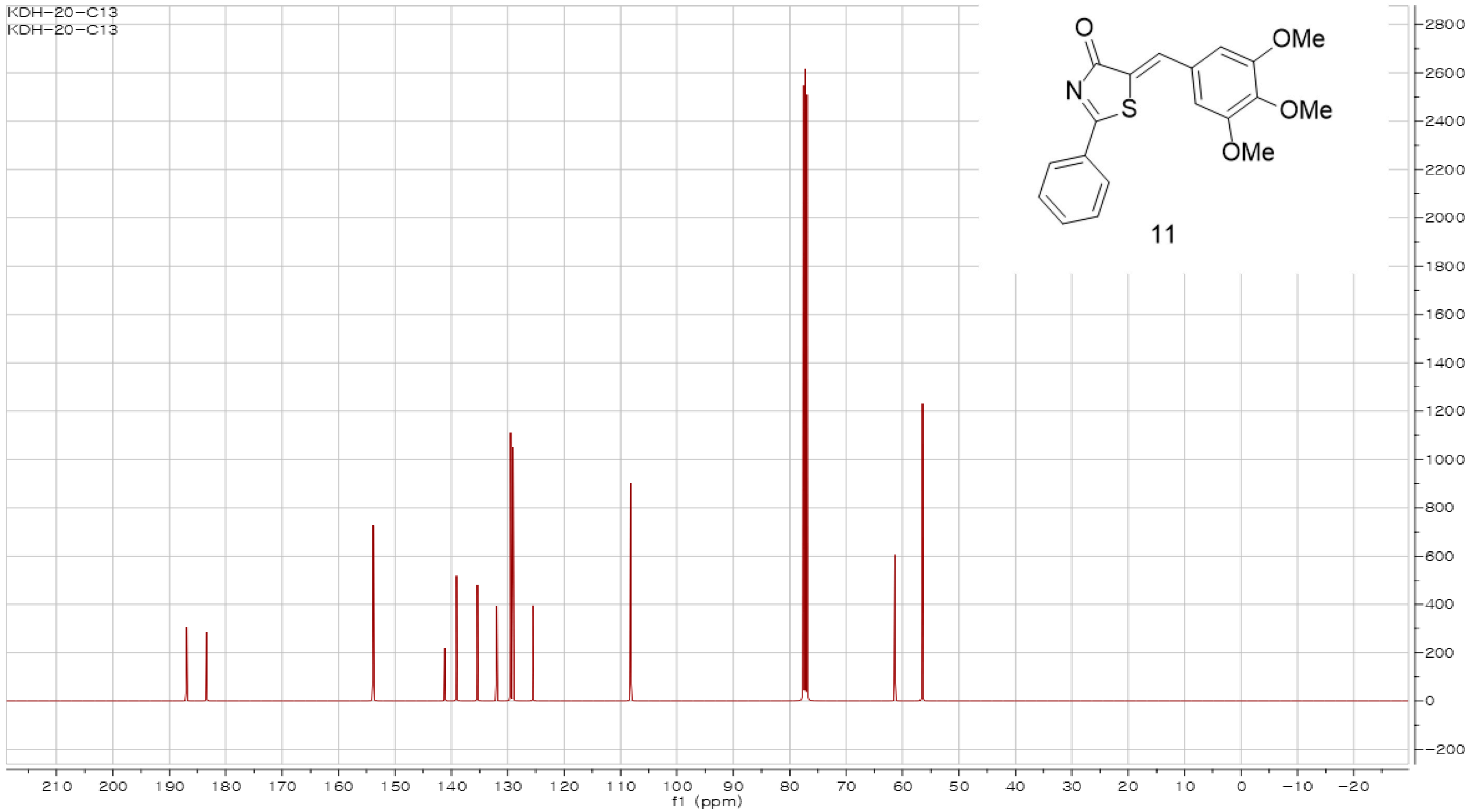

Figure S33. ^{13}C NMR spectrum of compound **11**.

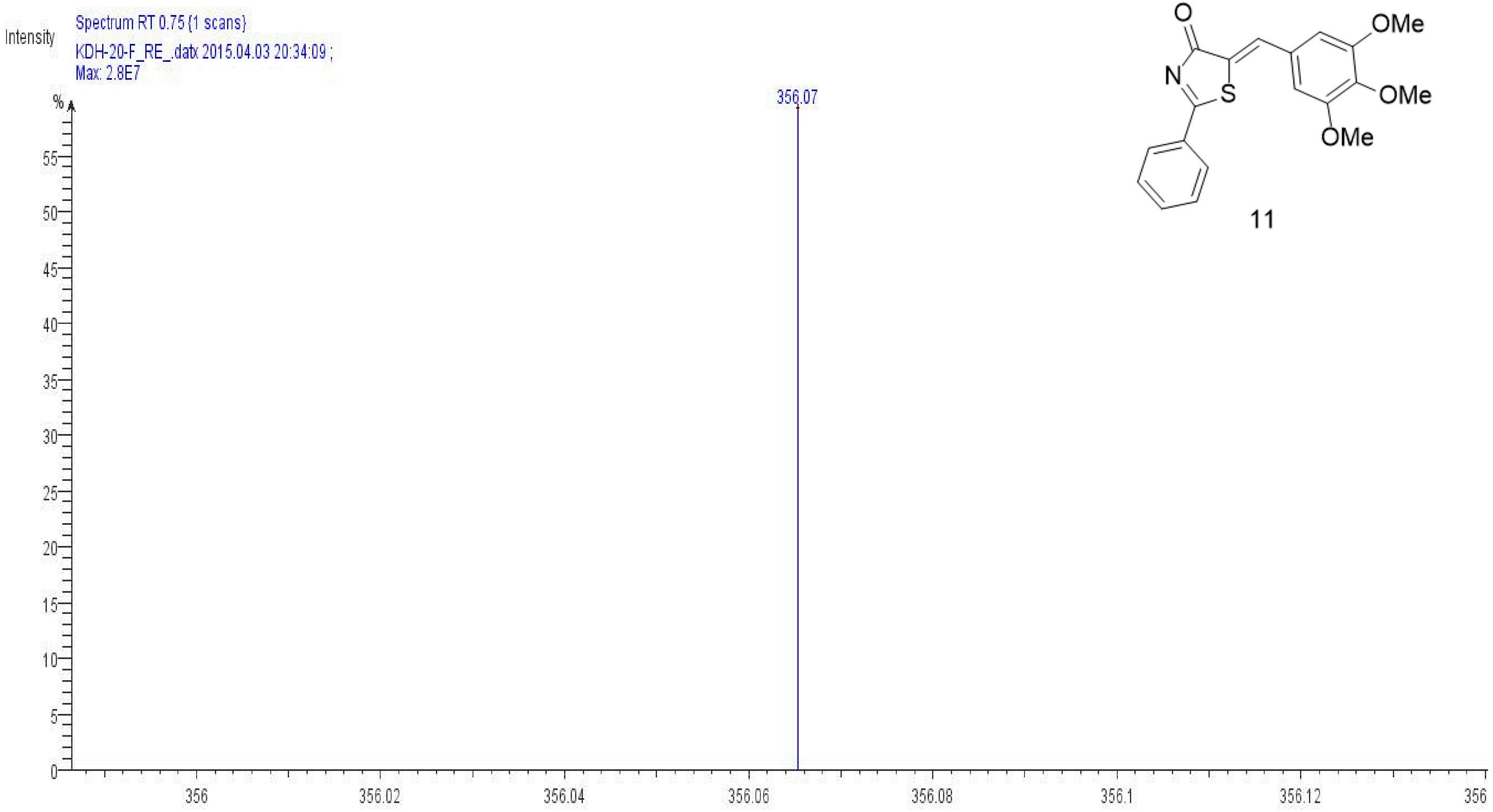

Figure S34. LRMS spectrum of compound **11**.

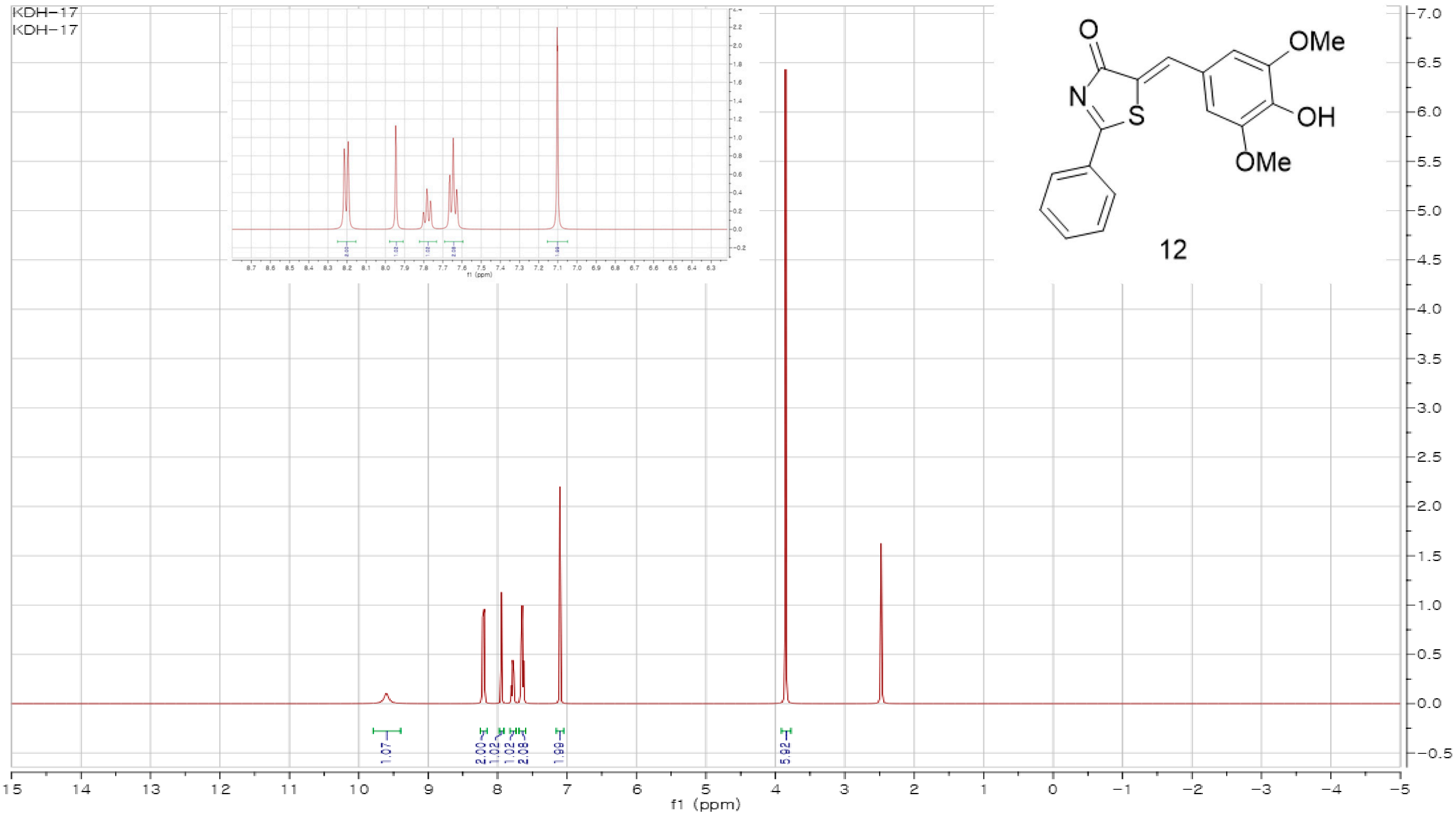

Figure S35. ^{1}H NMR spectrum of compound **12**.

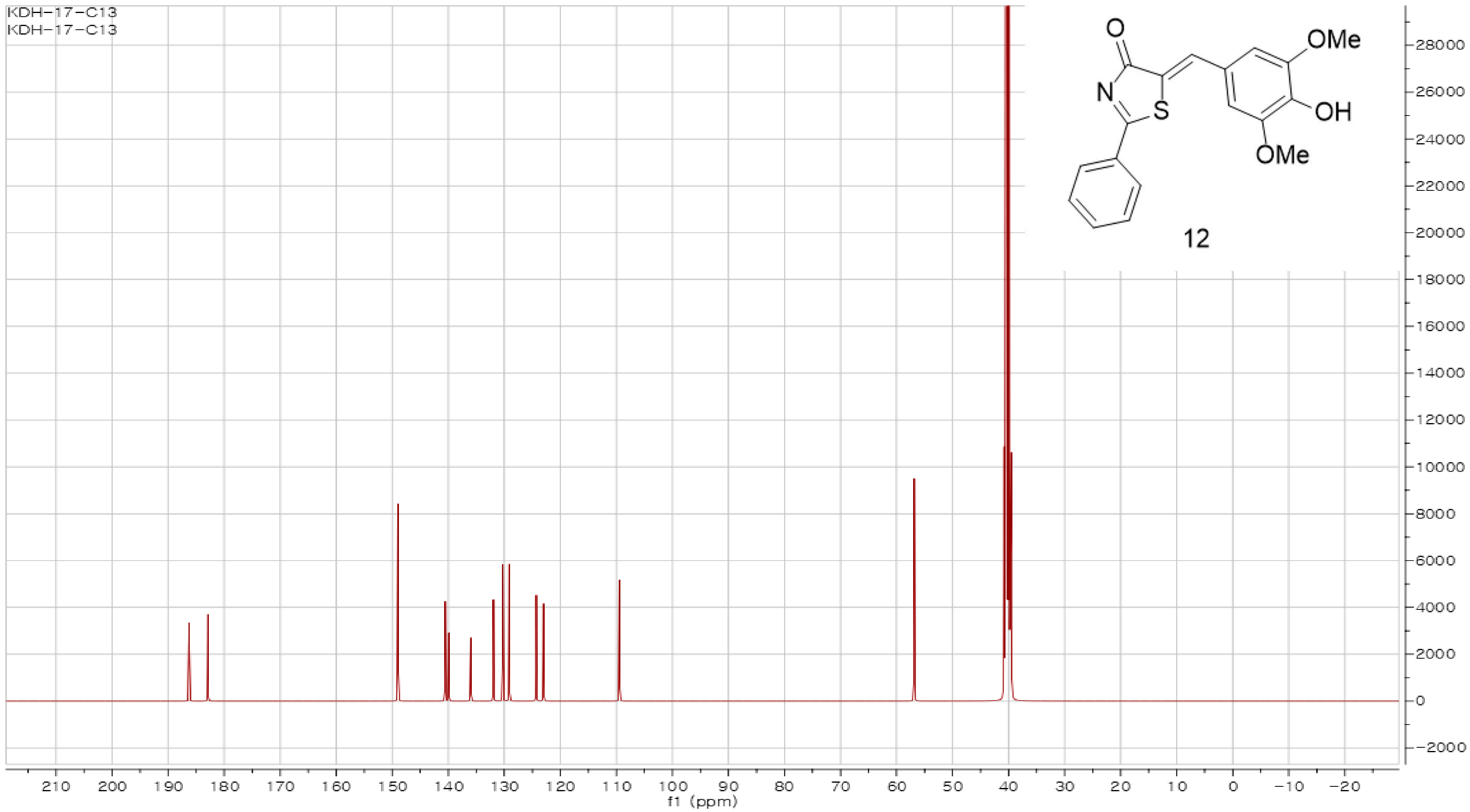

Figure S36. ^{13}C NMR spectrum of compound **12**.

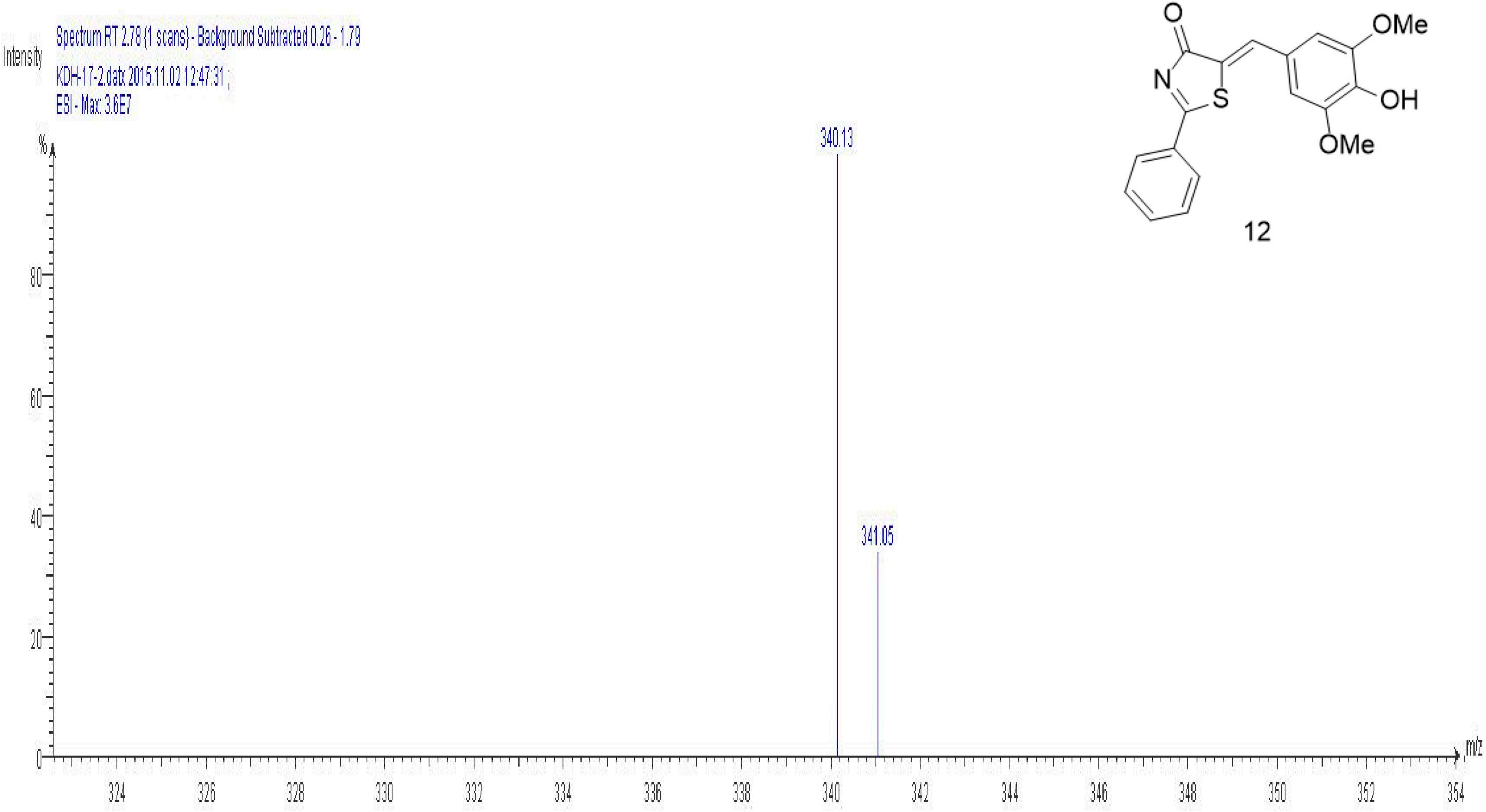

Figure S37. LRMS spectrum of compound **12**.

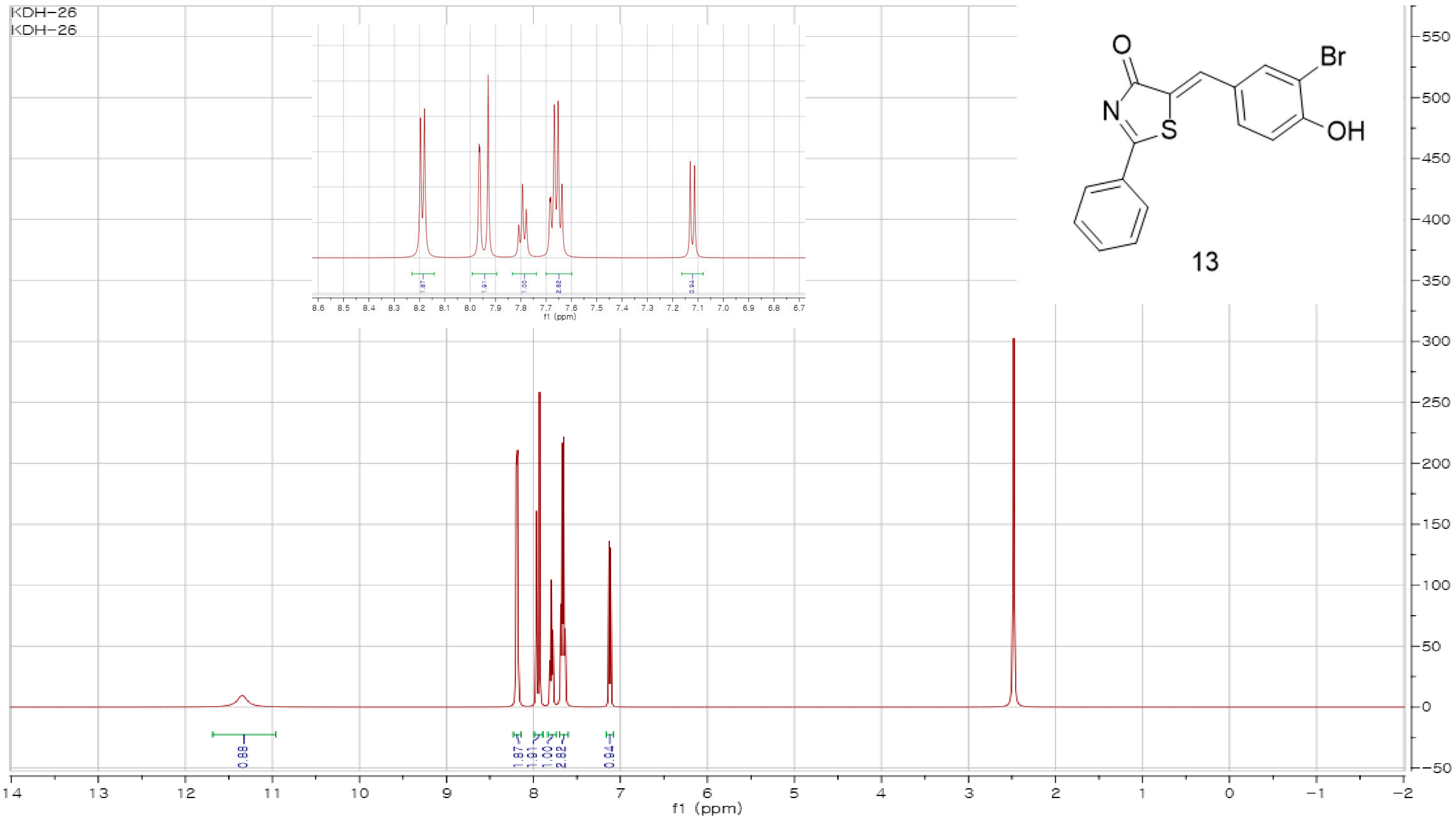

Figure S38. ^{1}H NMR spectrum of compound **13**.

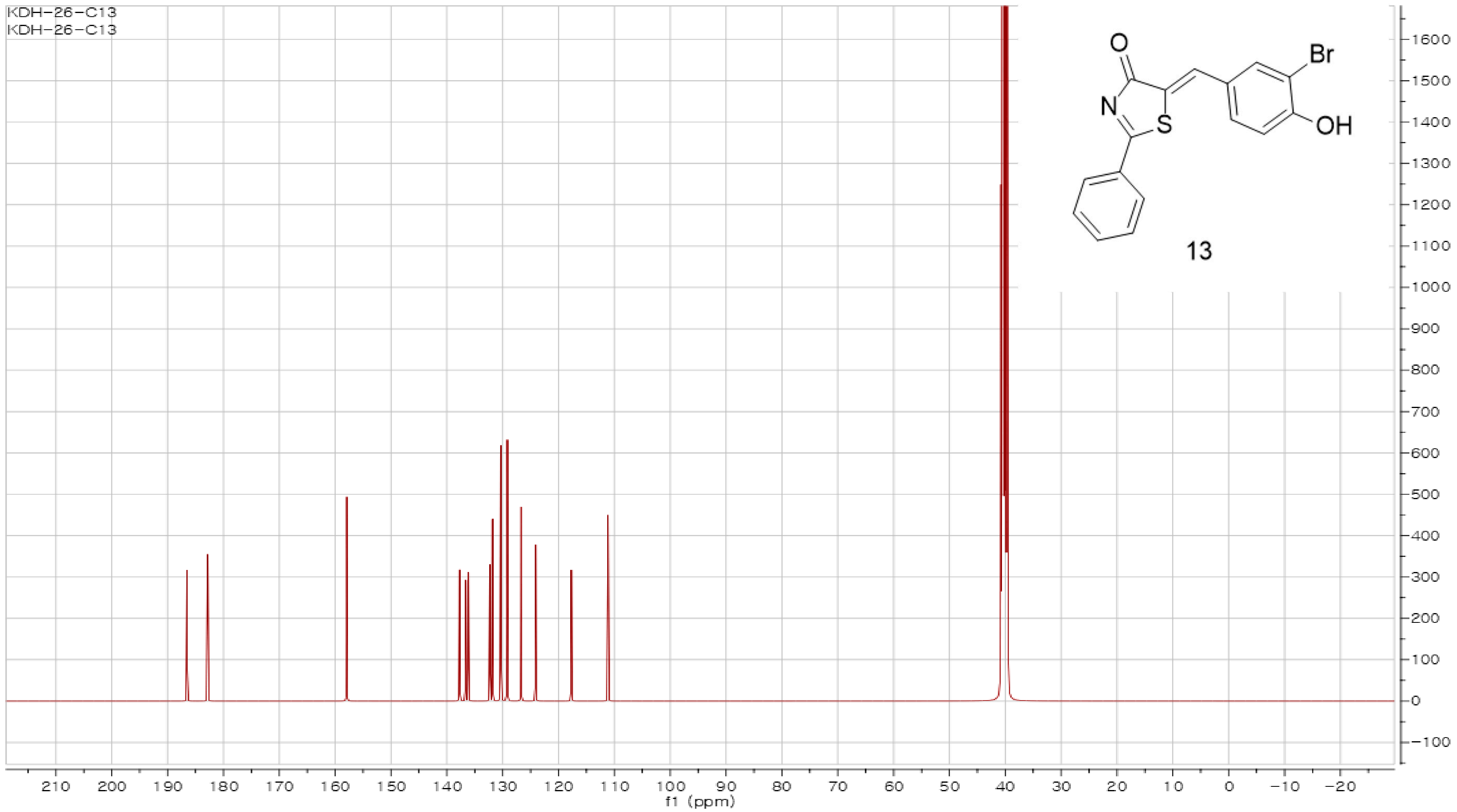

Figure S39. ^{13}C NMR spectrum of compound **13**.

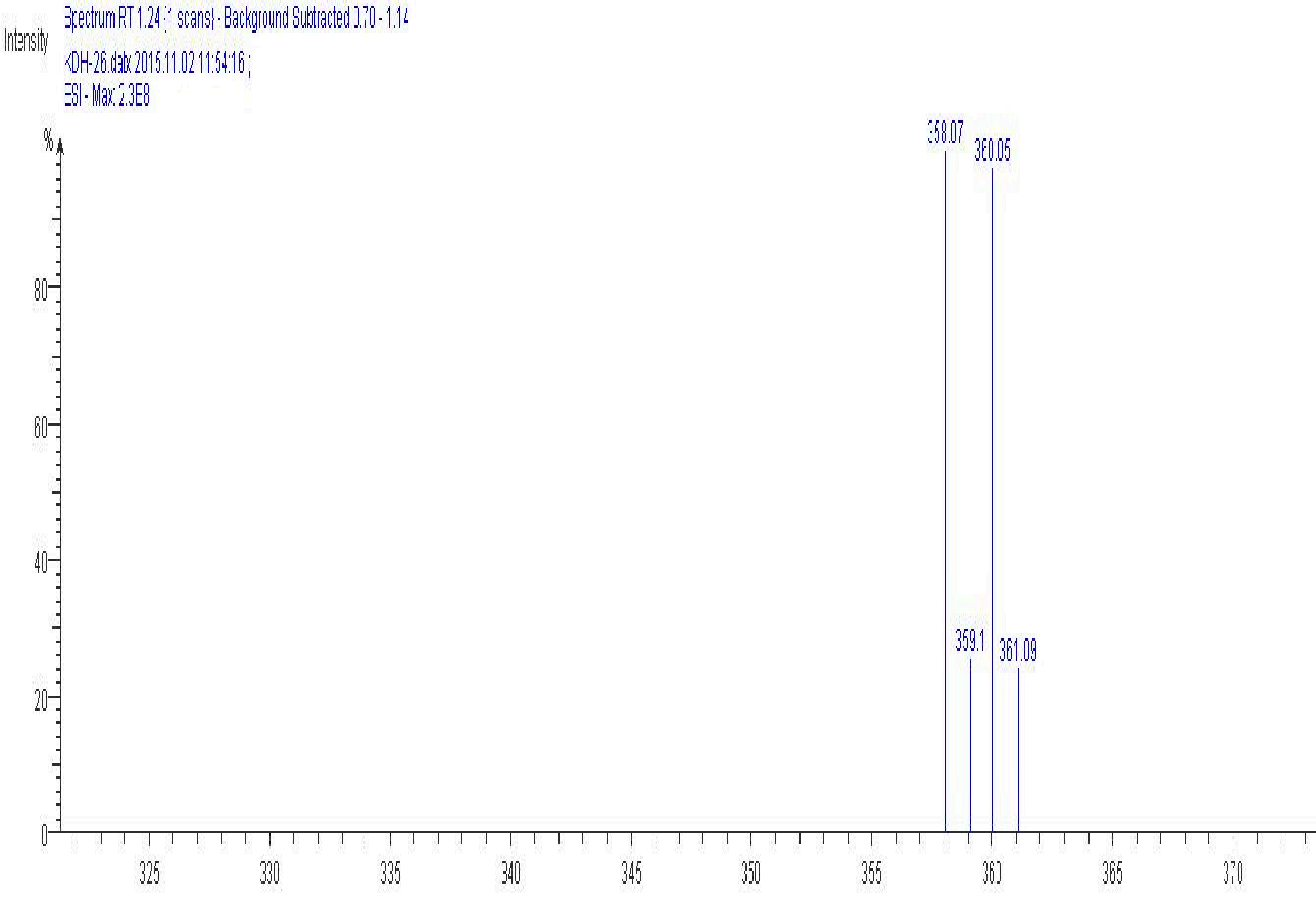

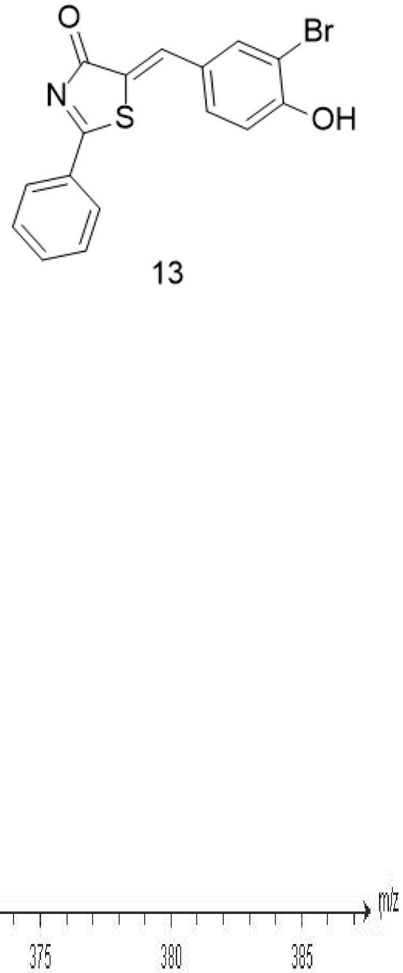

13

Figure S40. LRMS spectrum of compound **13**.

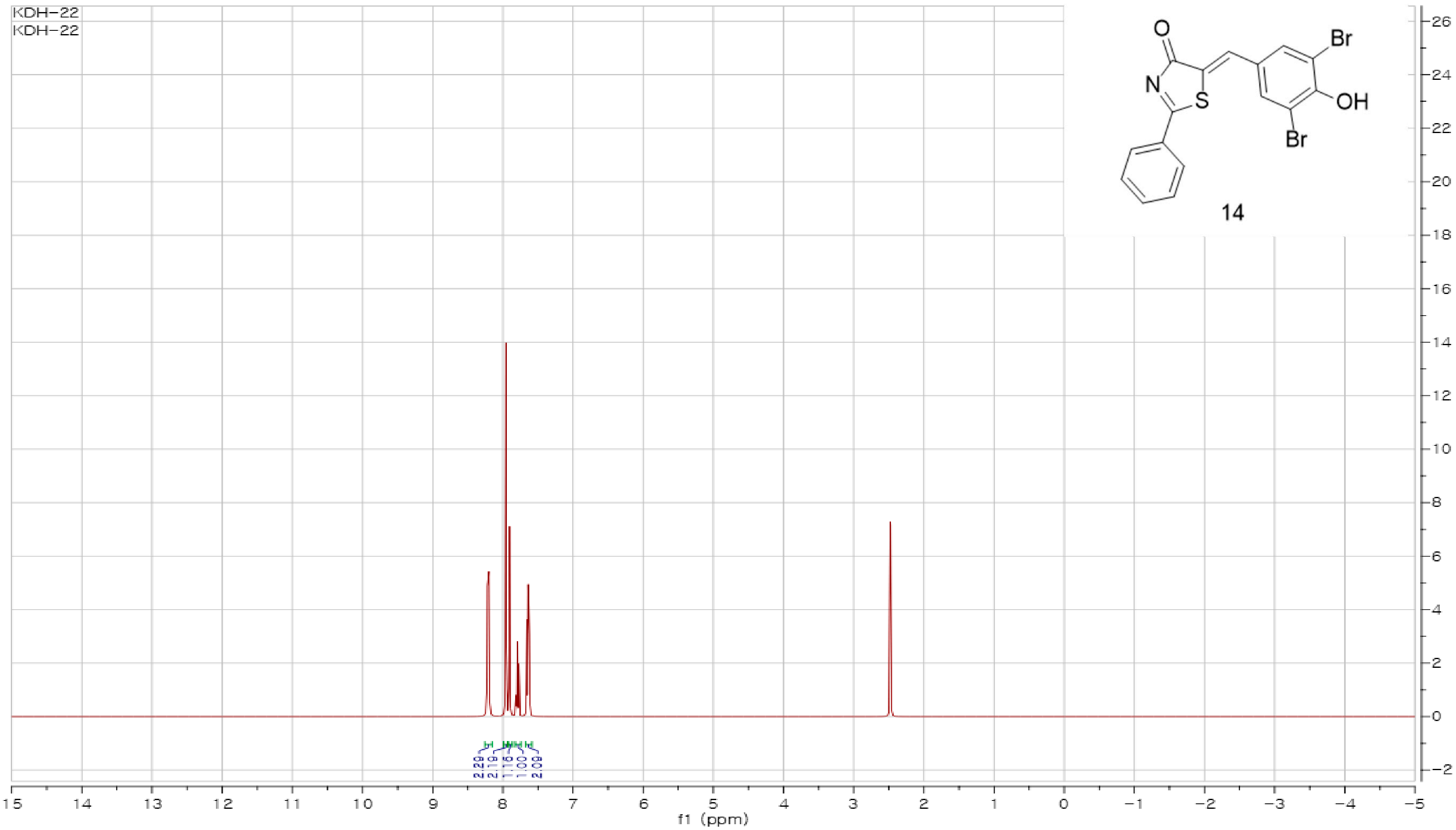

Figure S41. ^{1}H NMR spectrum of compound **14**.

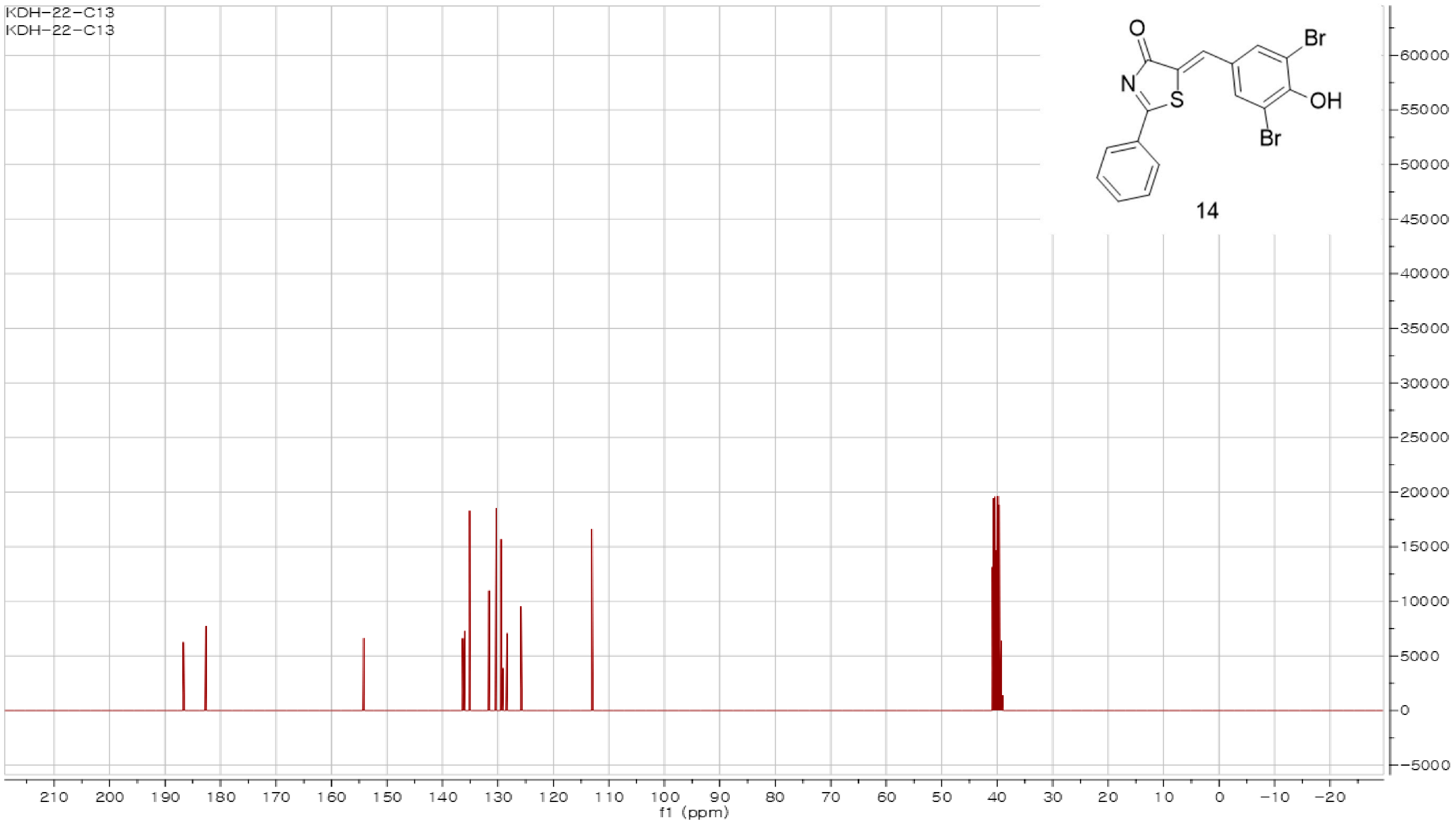

Figure S42. ^{13}C NMR spectrum of compound **14**.

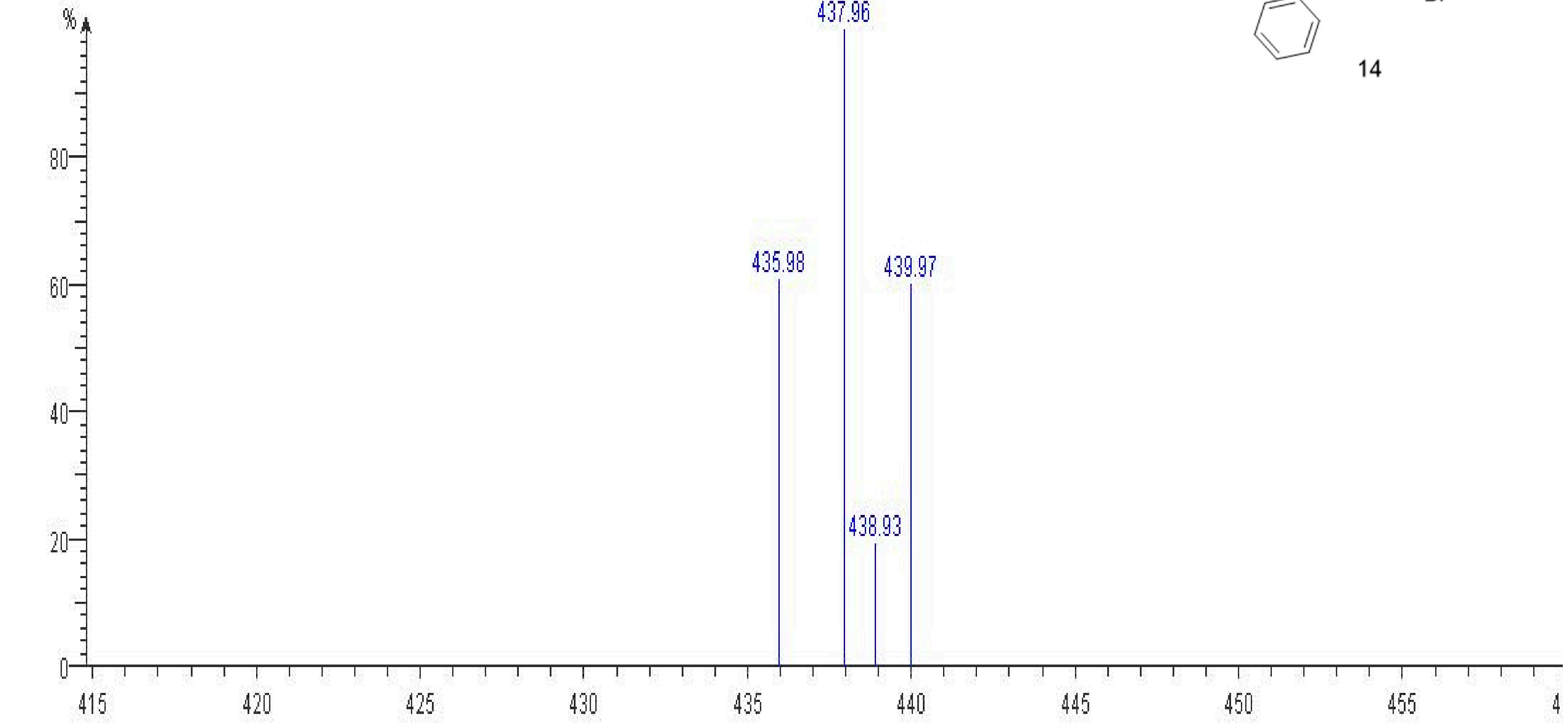

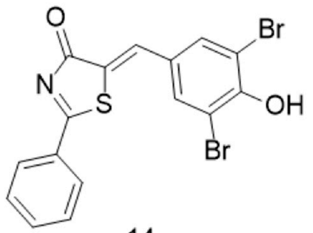

14

Figure S43. LRMS spectrum of compound **14**.

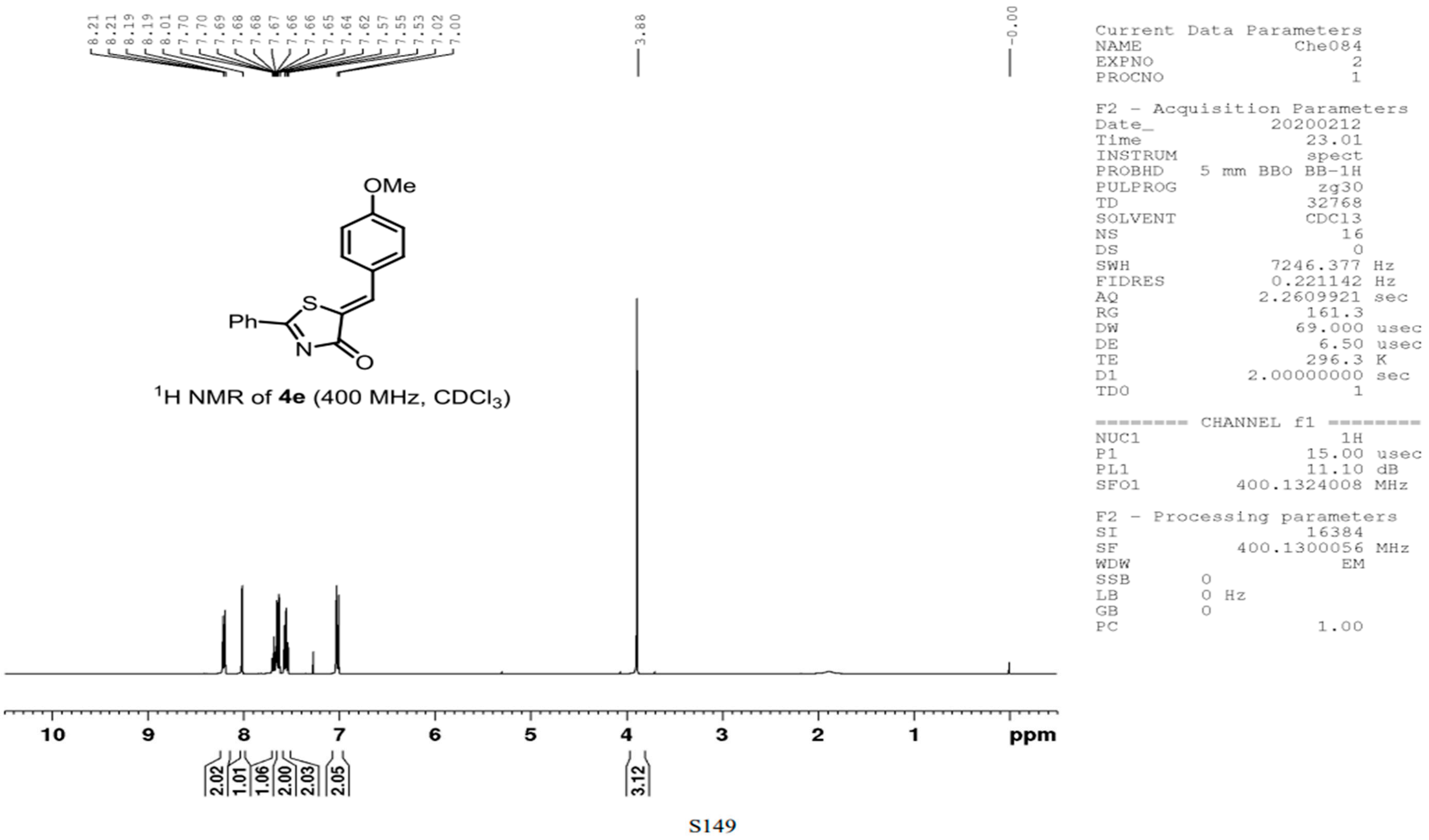

Figure S44. ^{1}H NMR spectrum of compound **8** reported in other article (*Org. Lett.* 2020, 22, 17, 6868–6872).

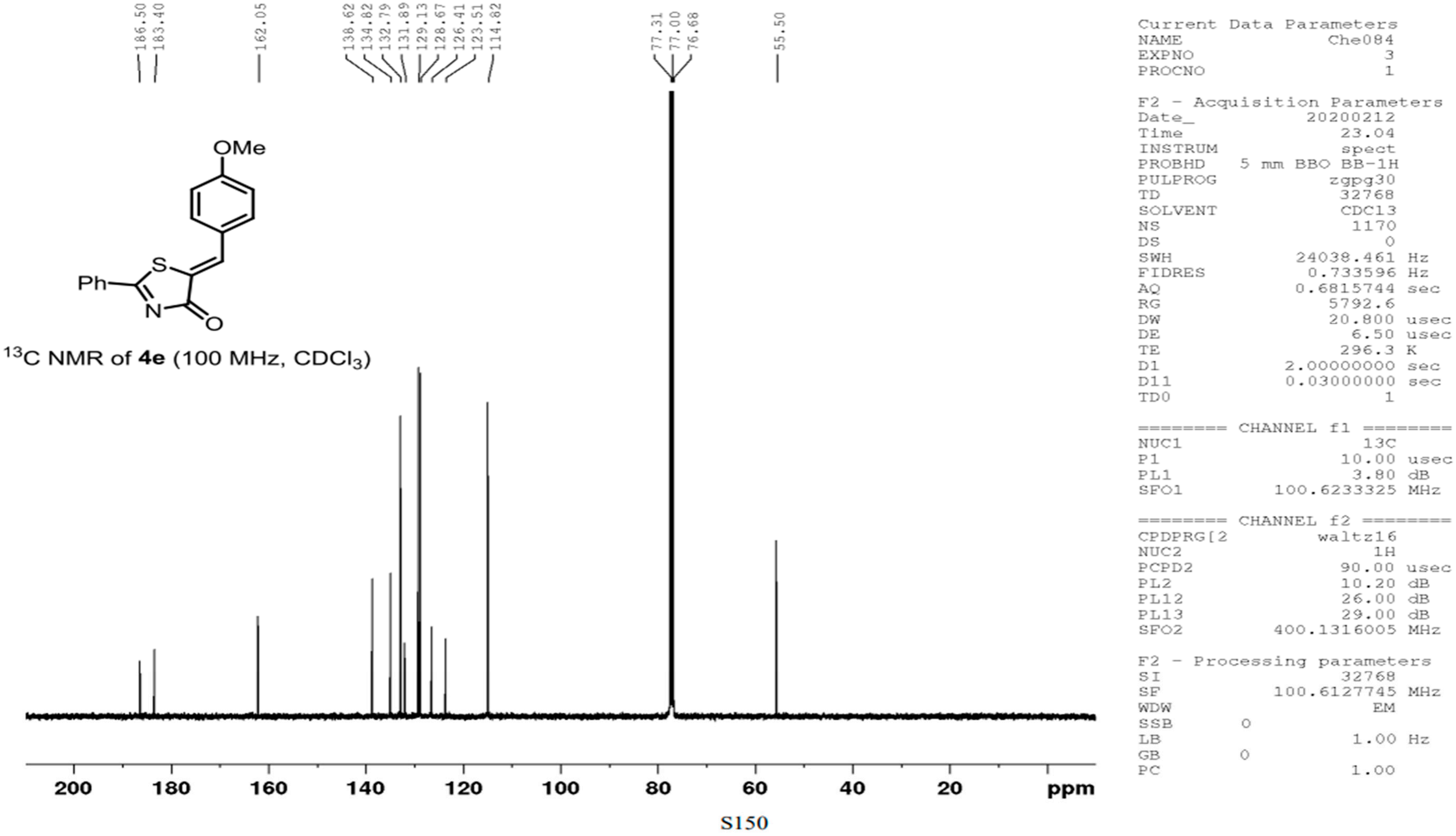

Figure S45. ^{13}C NMR spectrum of compound **8** reported in other article (*Org. Lett.* 2020, 22, 17, 6868–6872).

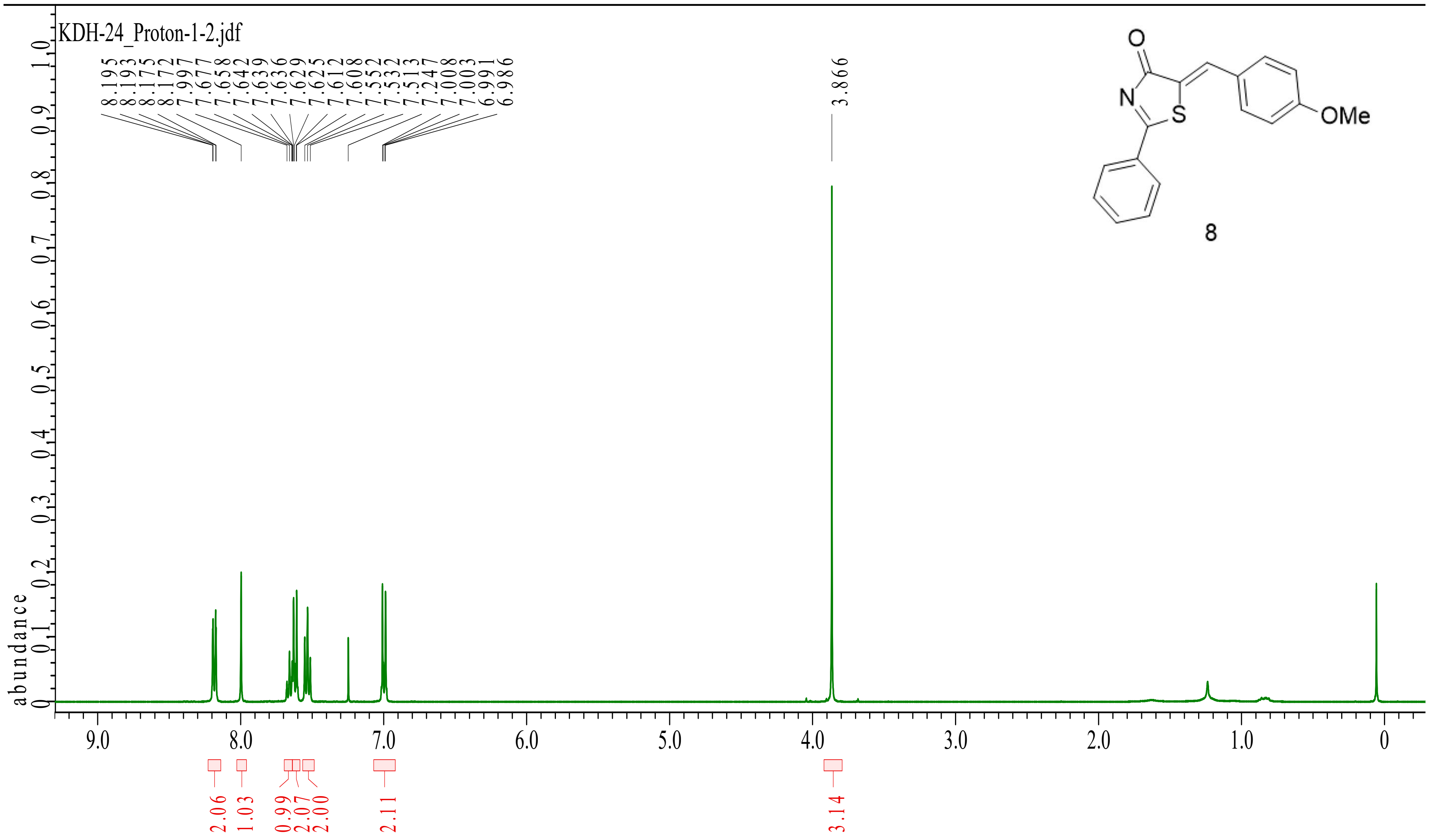

Figure S46. ^{1}H NMR spectrum of compound **8**.

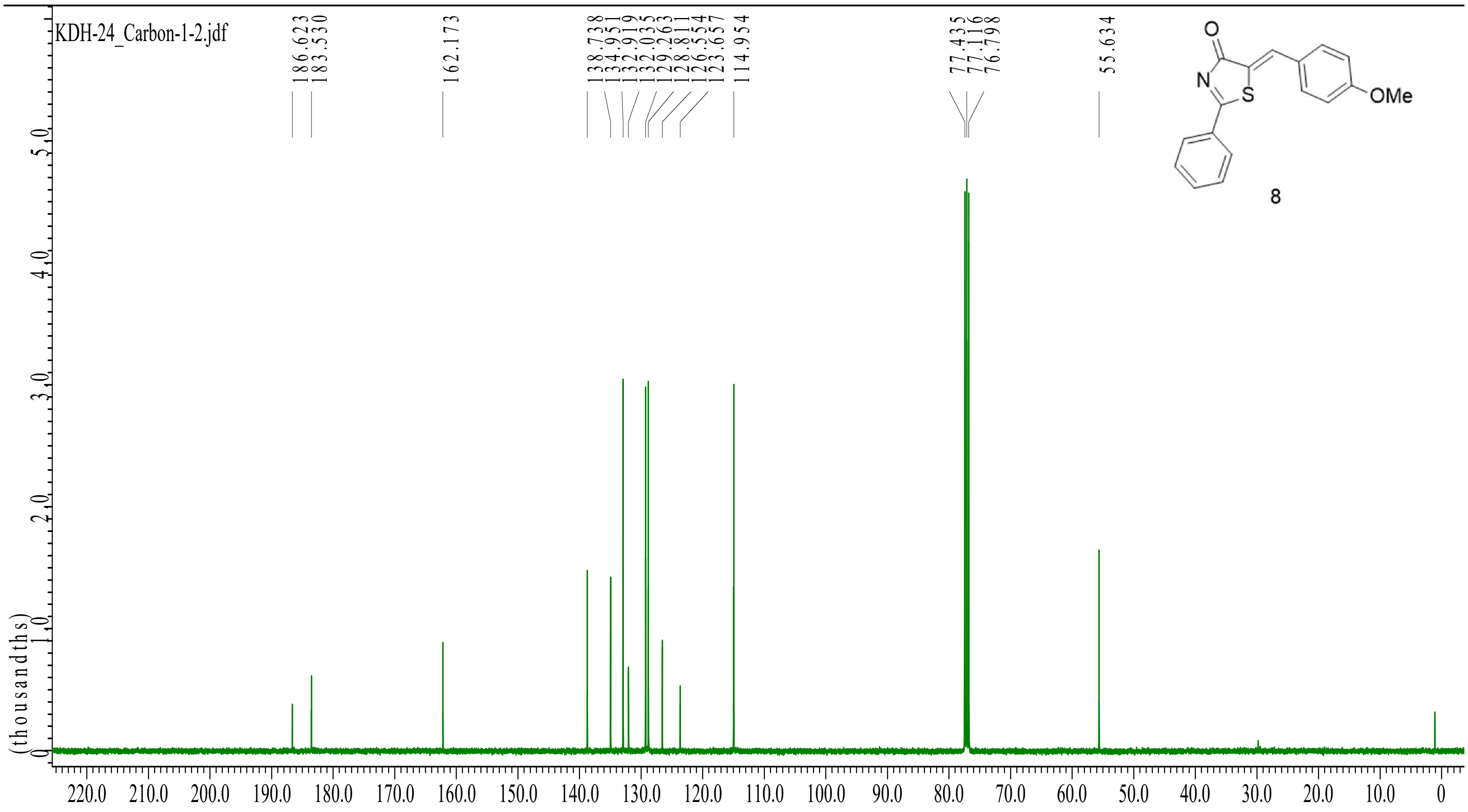

Figure S47. ^{13}C NMR spectrum of compound **8**.
